# Supplementary material for: Assessing the effectiveness of a 3-month day-and-night home closed-loop control combined with pump suspend feature compared with sensor-augmented pump therapy in youths and adults with suboptimally controlled type 1 diabetes: a randomised parallel study protocol
Source: BMJ Open. 2017 Jul 13;7(7):e016738. doi: 10.1136/bmjopen-2017-016738 (PMC5726132; doi:10.1136/bmjopen-2017-016738)
Supplement: Supplementary file 1 [file bmjopen-2017-016738supp001.pdf]

APPENDIX 1

Data Safety Monitoring Board

Dr Irl Hirsch (Chair), University of Washington School of Medicine, Seattle, WA, USA

Dr Michael Tansey (Member), University of Iowa Health Care, IA, USA

Dr Carol Levy (Member), Mount Sinai Hospital, New York, NY, USA

Study Management Team

Chief Investigator

Dr Roman Hovorka, University of Cambridge, Cambridge, UK

Principal Clinical Investigators (Youth)

Dr David Dunger, University of Cambridge, Cambridge, UK

Dr Daniela Elleri, Royal Hospital for Sick Children, Edinburgh, UK

Dr Fiona Campbell, St James's University Hospital, Leeds, UK

Dr Amy Criego, International Diabetes Center, Minneapolis, MN, USA

Principal Clinical Investigators (Adult)

Dr Mark Evans, University of Cambridge, Cambridge, UK

Dr Lalantha Leelarathna, Central Manchester University Hospitals NHS Foundation Trust, Manchester, UK

Dr Richard Bergenstal, International Diabetes Center, Minneapolis, MN, USA

Dr Viral Shah, University of Colorado Denver, Aurora, CO, USA

Clinical Investigators

Dr Carlo L Acerini, University of Cambridge, Cambridge, UK

Dr Hood Thabit, University of Cambridge, Cambridge, UK

Dr Martin Tauschmann, University of Cambridge, Cambridge, UK

Dr Lia Bally, University of Cambridge, Cambridge, UK

Study Co-ordinator (UK)

Mrs Josephine Hayes, University of Cambridge, Cambridge, UK

Study Co-ordinator (USA)

Dr Roy Beck, Jaeb Center for Health Research, Tampa, FL, USA

Medical Sociologist

Dr Julia Lawton, University of Edinburgh, Edinburgh, UK

Study Statistician

Dr Craig Kollman, Jaeb Center for Health Research, Tampa, FL, USA

Study Data Manager

Carlos Murphy, Jaeb Center for Health Research, Tampa, FL, USA

Study Sponsor (UK)

Cambridge University Hospitals NHS Foundation Trust, jointly with University of Cambridge, UK

Study Sponsor (USA)

Jaeb Center for Health Research, Tampa, FL, USA

Funder

JDRF

UNIVERSITY OF CAMBRIDGE

Cambridge University Hospitals NHS
NHS Foundation Trust

IMS Institute of Metabolic Science

Patient ID Label:

Centre NRES number: ....

Patient identification number for this trial: …………………….

CONSENT FORM

(Adults and Youth aged 16 and over)

Title of Project: Home testing of day and night closed loop with pump suspend feature

Name of Researcher: ....

Please initial box

1. I confirm that I have read and understood the information sheet version 2.1 dated 17/11/2015 for the above study and have had the opportunity to consider the information and I am satisfied with the answers I have been given. ☐

2. I understand that my participation is voluntary and that I am free to withdraw at any time, without giving any reason, without my medical care or legal rights being affected in any way.

3. I give consent for the taking of blood and urine samples.

4. If I want to change my mind in the future and withdraw my consent for this study, then I understand that, if I request it, the samples will be destroyed.

5. I give consent to my GP being informed of my participation in this study.

6. I give consent to the sharing of anonymised research data arising from this study with other study partners in Europe and USA.

7. I give consent to anonymised blood samples being sent to USA for analysis.

8. If I choose to communicate with the research team via email, I acknowledge the associated risks as outlined in the information sheet.

9. I understand that relevant sections of my medical notes and data collected during the study may be looked at by individuals from the NHS Trust and regulatory bodies for audit or monitoring purposes.

10. If randomised to the closed loop study arm:

    a. I agree to participate in two qualitative interviews and for my contact details to be given to the study psychologist.

    b. I agree to have my family member/partner contacted by the study psychologist and to have their contact details forwarded.

    c. I agree for my interviews to be digitally recorded.

    d. I understand that anonymised quotes may be used in reports and publications.

11. I agree to return the study devices at the end of the study or earlier if consent is withdrawn.

12. I understand that in the unlikely event that study devices are not returned, legal measures may be undertaken, as a last resort after all other options have been exhausted, to recover the study devices.

13. I agree to take part in the study, and to follow the study team's instructions for the purposes of the study.

14. I give consent for my personal data to be kept by the clinical research team for up to 5 years after termination of the study so that they may contact me in the future with results of this and other similar studies. ☐

Name of participant	Date	Signature
Name of Person taking consent (if different from researcher)	Date	Signature
Researcher	Date	Signature

Copies: *1 for volunteer; 1 for researcher; 1 to be kept with hospital notes*
