# Supplementary material for: Assessing the effectiveness of a 3-month day-and-night home closed-loop control combined with pump suspend feature compared with sensor-augmented pump therapy in youths and adults with suboptimally controlled type 1 diabetes: a randomised parallel study protocol
Source: BMJ Open. 2017 Jul 13;7(7):e016738. doi: 10.1136/bmjopen-2017-016738 (PMC5726132; doi:10.1136/bmjopen-2017-016738)
Supplement: Author's manuscript [file bmjopen-2017-016738.draft_revisions.pdf]

Assessing the effectiveness of 3 months day-and-night home closed-loop control combined with pump suspend feature compared to sensor augmented pump therapy in youths and adults with sub-optimally controlled type 1 diabetes: a randomised parallel study protocol

Journal:	*BMJ Open*
Manuscript ID	bmjopen-2017-016738
Article Type:	Protocol
Date Submitted by the Author:	06-Mar-2017
Complete List of Authors:	Bally, Lia; University of Cambridge, Wellcome-Trust MRC Institute of Metabolic Science; Cambridge University Hospitals NHS Foundation Trust<br>Thabit, Hood; University of Cambridge, Wellcome Trust-MRC Institute of Metabolic Science; Cambridge University Hospitals NHS Foundation Trust<br>Tauschmann, Martin; University of Cambridge; University of Cambridge, Department of Paediatrics<br>Allen, Janet; University of Cambridge, Wellcome Trust-MRC Institute of Metabolic Science; University of Cambridge, Department of Paediatrics<br>Hartnell, Sara; Cambridge University Hospitals NHS Foundation Trust<br>Wilinska, Malgorzata; University of Cambridge, Wellcome Trust-MRC Institute of Metabolic Science; University of Cambridge, Department of Paediatrics<br>Exall, Jane; Leeds Children's Hospital<br>Huegel, Viki; Jaeb Center for Health Research<br>Sibayan, Judy; Jaeb Center for Health Research<br>Borgman, Sarah; Jaeb Center for Health Research<br>Cheng, Peiyao; Jaeb Center for Health Research<br>Blackburn, Maxine; University of Edinburgh, Centre for Population Health Sciences<br>Lawton, Julia; University of Edinburgh, Centre for Population Health Sciences<br>Elleri, Daniela; Royal Hospital for Sick Children<br>Leelarathna, Lalantha; Central Manchester University Hospitals NHS Foundation Trust, Manchester Academic Health Science Centre, Manchester Diabetes Centre<br>Acerini, Carlo; University of Cambridge, Department of Paediatrics<br>Campbell, Fiona; Leeds Children's Hospital<br>Shah, Viral; University of Colorado, Barbara Davis Center for Diabetes<br>Criego, Amy; International Diabetes Center at Park Nicollet<br>Evans, Mark; University of Cambridge, Wellcome Trust-MRC Institute of Metabolic Science; Cambridge University Hospitals NHS Foundation Trust<br>Dunger, David; University of Cambridge, Department of Paediatrics<br>Kollman, Craig; Jaeb Center for Health Research<br>Bergenstal, Richard; International Diabetes Center at Park Nicollet<br>Hovorka, Roman; University of Cambridge, Wellcome Trust-MRC Institute of Metabolic Science; University of Cambridge, Department of Paediatrics
Primary Subject	Diabetes and endocrinology

Heading:	
Secondary Subject Heading:	Medical management
Keywords:	type 1 diabetes, closed-loop, artificial pancreas

SCHOLARONE™ Manuscripts

For peer review only

Assessing the effectiveness of 3 months day-and-night home closed-loop control combined with pump suspend feature compared to sensor augmented pump therapy in youths and adults with sub-optimally controlled type 1 diabetes: a randomised parallel study protocol

[1,2]Bally L, [1,2]Thabit H, [1,3]Tauschmann M, [1,3]Allen JM, [2]Hartnell S, [1,3]Wilinska ME, [4]Exall J, [5]Huegel V, [5]Sibayan J, [5]Borgman S, [5]Cheng P, [6]Blackburn M, [6]Lawton J, [7]Elleri D, [8]Leelarathna L, [1,3]Acerini CL, [4]Campbell F, [9]Shah VN, [10]Criego A, [1,2]Evans ML, [1,3]Dunger DB, [5]Kollman C, [10]Bergenstal RM, and [1,3]Hovorka R

[1]Wellcome Trust-MRC Institute of Metabolic Science, University of Cambridge, Cambridge, UK

[2]Department of Diabetes & Endocrinology, Cambridge University Hospitals NHS Foundation Trust, Cambridge, UK

[3]Department of Paediatrics, University of Cambridge, Cambridge, UK

[4]Leeds Children's Hospital, Leeds, UK

[5]Jaeb Center for Health Research, Tampa, FL, USA

[6]Centre for Population Health Sciences, University of Edinburgh, Edinburgh, UK

[7]Royal Hospital for Sick Children, Edinburgh, UK

[8]Manchester Diabetes Centre, Central Manchester University Hospitals NHS Foundation Trust, Manchester Academic Health Science Centre, Manchester, UK

[9]Barbara Davis Center for Diabetes, University of Colorado Denver, Aurora, CO, USA

[10]International Diabetes Center at Park Nicollet, MN, USA

Correspondence

Roman Hovorka, PhD

University of Cambridge Metabolic Research Laboratories and NIHR Cambridge Biomedical Research Centre,

Wellcome Trust-MRC Institute of Metabolic Science, Box 289,

Addenbrooke's Hospital, Hills Road, Cambridge CB2 0QQ, UK

tel: +44 1223 762 862, fax: +44 1223 330 598, e-mail: rh347@cam.ac.uk

Key Words: Type 1 diabetes, Closed-loop, Artificial pancreas

Word count abstract: 287

Word count paper: 4924

For peer review only

ABSTRACT

Introduction Despite therapeutic advances, many individuals with type 1 diabetes are unable to achieve tight glycaemic target without increasing the risk of hypoglycaemia. The objective of the present study is to determine the effectiveness of 3-month day-and-night home closed-loop glucose control combined with a pump suspend feature, compared to sensor-augmented insulin pump therapy in youths and adults with sub-optimally controlled type 1 diabetes.

Methods and analysis The study adopts an open-label, multi-centre, multi-national (UK and USA), randomised, single-period, parallel design and aims for 84 randomised patients. Participants are youths (6-21 years) or adults (>21 years) with type 1 diabetes treated with insulin pump therapy and sub-optimal glycaemic control [glycated haemoglobin (HbA1c) ≥7.5% (58mmol/mol) and ≤10% (86mmol/mol)]. Following a 4-week run-in period, eligible participants will be randomised to 3-month use of automated closed-loop insulin delivery combined with pump suspend feature, or to sensor-augmented insulin pump therapy. Analyses will be conducted on an intention to treat basis. The primary outcome is the time spent in the target glucose range from 3.9 to 10.0mmol/l based on continuous glucose monitoring levels during the 3-month free living phase. Secondary outcomes include HbA1c at 3 months, mean glucose, time spent below and above target; time with glucose levels <3.5mmol/l and <2.8mmol/l; area under the curve when sensor glucose is <3.5mmol/l, time with glucose levels >16.7mmol/l, glucose variability; total, basal and bolus insulin dose, change in body weight. Participants' and their families' perception in terms of lifestyle change, daily diabetes management and fear of hypoglycaemia will be evaluated.

Ethics and dissemination Ethics/institutional review board approval has been obtained. Before screening, all participants/guardians will be provided with oral and written information about the trial. The study will be disseminated by peer-review publications and conference presentations.

Trial registration: NCT025231131 (ClinicalTrials.gov)

For peer review only

Strengths and limitations of the study

Strengths

- The study is a multi-centre multi-national randomised parallel design study comparing automated glucose control combined with pump suspend feature to sensor-augmented insulin pump therapy
- The study includes youth and adult participants across wide range age groups and geographical locations
- Participants in both study arms have equal numbers of study visits
- Psychosocial evaluation will be undertaken with participants and family members using in-depth interviews and validated questionnaires to determine the acceptability of intervention, quality of life and diabetes management

Limitations

- The study excludes participants who are hypoglycaemia unaware and living alone
- The study is open-label and the comparator does not include the use of pump suspend feature
- The study does not include participants who have HbA1C below 7.5% and above 10%

For peer review only

Introduction

Type 1 diabetes mellitus (T1D) is caused by an immune-mediated destruction of insulin-producing pancreatic ß cells resulting in insulin deficiency[1]. It commonly presents in childhood, but one fourth of cases are diagnosed in adults. T1D accounts for 5-10% of the total diabetes cases worldwide[2]. The incidence of T1D is still increasing by around 3% every year, particularly among children[2]. Around 86,000 children develop T1D each year and in 2015 the number of children with T1D exceeded half a million for the first time[2].

Despite rapid advancements in insulin delivery and the on-going development of more physiological insulin preparations[3], achieving optimal glycaemic control while avoiding hypoglycaemia remains a challenge for many people with T1D. The emergence of continuous glucose monitoring (CGM) over the last decade[4,5], which enables users to view real-time interstitial glucose readings and receive alarms for impending hypo- or hyperglycaemia facilitating appropriate changes in insulin therapy, is a major step towards improved diabetes monitoring. Studies have shown clinical benefit of CGM in reducing HbA1c with regular us of the device[6]. Sensor-augmented pumps (SAP) combine real-time CGM with insulin pump therapy and studies have shown HbA1c reduction compared to multiple daily injection basal-bolus therapy[7]. Automatic suspension of insulin delivery by the pump when a predefined threshold is reached (low glucose suspend feature) has been demonstrated to significantly reduce the burden of hypoglycaemia[8].

The development of a closed-loop system that combines glucose monitoring with computer-based algorithm directed insulin delivery[9] may provide further improvements in glycaemic control while reducing hypoglycaemia[10,11] and represents an emerging treatment option for people with T1D[12]. Closed-loop differs from conventional pump therapy, characterised by pre-programmed basal delivery, through the use of a control algorithm which directs subcutaneous insulin delivery according to sensor glucose levels. People with T1D are known to have significant variability in daily insulin requirements[13], which may be addressed by closed-loop.

Closed-loop studies have progressed from fully supervised research facility settings[14,15], to remotely monitored transitional settings such as diabetes camp and hotel settings[16,17]. The use of closed-loop during unsupervised free living over extended periods represents the ultimate testing bed within the target environment (Table 1). The longest randomised day-and-night home study to date have shown benefit in glycaemic control with concurrent reduction of the risk of hypoglycaemia[10]. Based on a recent non-randomised 3-month pivotal study[12], the US Food and Drug Agency (FDA) approved the insulin-only closed-loop system for use in clinical practice[18].

No study thus far has combined the pump suspend feature[8] with closed-loop insulin delivery. In previous closed-loop systems, pre-programmed basal insulin rates are delivered in the event of communication failure between control algorithm device and the insulin pump. The addition of pump suspend feature to a closed-loop system further increases user safety by discontinuing insulin delivery at low sensor glucose levels.

We plan to assess the efficacy, safety, utility and acceptability of 3-month day-and-night home closed-loop glucose control combined with pump suspend feature in youths and adults under free-living conditions compared to sensor-augmented pump therapy.

For peer review only

Methods and Analysis

Overview

This is an open-label, multi-centre, multi-national, randomised, one-period, parallel study contrasting day-and-night automated closed-loop glucose control combined with pump suspend feature, to sensor augmented insulin pump therapy during free-living home setting (Figure 1, protocol version 5.0 dated 28 February 2017). Study participants will include youths and adults with sub-optimally controlled T1D treated with insulin pump therapy. Recruited participants will be randomly assigned to 3 months of study intervention. The study will aim to randomise 84 participants stratified at each study centre according to the two age groups [equal proportions of youths (6-21 years), and adults (22 years and older)]. The University of Cambridge (UK) and Jaeb Center (USA) will be the coordinating centres. Clinical sites include:

1) Addenbrooke's Hospital, Cambridge, UK (adults and youths)
2) Royal Hospital for Sick Children, Edinburgh, UK (youths)
3) Leeds Teaching Hospital, Leeds, UK (youths)
4) Manchester Royal Infirmary, Manchester, UK (adults)
5) International Diabetes Center at Park Nicollet, Minneapolis, USA (adults and youths)
6) Barbara Davis Center for Childhood Diabetes, Aurora, USA (adults)

Qualitative interviews will be carried by University of Edinburgh, UK. Written informed consent/assent will be obtained from all participants and guardians, as appropriate, before any study related activities.

Inclusion criteria

- At least 6 years or older [with equal proportion of youths (6 to 21 years) and adults (22 years and older)]
- T1D as defined by WHO for at least 1 year or confirmed C-peptide negative
- An insulin pump user for at least 3 months, with good knowledge of insulin self-adjustment as judged by the investigator
- Treated with one of the U-100 rapid acting insulin analogues only (insulin aspart, lispro but not glulisine)
- Willing to perform regular capillary blood glucose monitoring, with at least 4 blood glucose measurements taken every day
- Screening HbA1c ≥ 7.5% (58.5mmol/mol) and ≤ 10% (86mmol/mol) based on analysis from local laboratory or equivalent
- Literate in English
- Willing to wear CGM
- Willing to wear closed-loop system at home
- Willing to follow study specific instructions
- Willing to upload pump and CGM data at regular intervals
- Willing to restrict alcohol consumption to ≤ 2 units per day throughout the study period
- Female participants of child bearing age should be on effective contraception and must have a negative urine-HCG pregnancy test at screening
- Living with someone who is trained to administer intramuscular glucagon and is able to seek emergency assistance
- Access to Wi-Fi at home

For peer review only

Exclusion criteria

- Non-type 1 diabetes mellitus including those secondary to chronic disease
- Using real-time CGM on regular basis in preceding 3 months
- Any other physical or psychological disease likely to interfere with the normal conduct of the study and interpretation of the study results as judged by the investigator
- Untreated coeliac disease, adrenal insufficiency, or hypothyroidism
- Current treatment with drugs known to interfere with glucose metabolism, e.g. systemic corticosteroids, non-selective beta-blockers and MAO inhibitors etc.
- Known or suspected allergy to insulin
- Clinically significant nephropathy (estimated glomerular filtration rate< 45ml/min) or on dialysis, neuropathy or active retinopathy (defined as presence of maculopathy or proliferative changes) as judged by the investigator
- Adults: one or more episodes of severe hypoglycaemia as defined by American Diabetes Association[19] in preceding 6 months; youths: one or more episodes of severe hypoglycaemia during the previous 6 months (adults and adolescents: severe hypoglycaemia is defined as an event requiring assistance of another person to actively administer carbohydrates, glucagon, or take other corrective actions including episodes of hypoglycaemia severe enough to cause unconsciousness, seizures or attendance at hospital; children: severe hypoglycaemia is defined as an event associated with a seizure or loss of consciousness)
- Random C-peptide > 100pmol/l with concomitant blood glucose >4mmol/l (72mg/dl)
- Regular use of acetaminophen
- Lack of reliable telephone facility for contact
- Total daily insulin dose ≥ 2IU/kg/day
- Total daily insulin dose < 15IU/day
- Pregnancy, planned pregnancy, or breast feeding
- Severe visual impairment
- Severe hearing impairment
- Significantly reduced hypoglycaemia awareness in participants 18 year and older defined by Gold score >4
- Using implanted internal pace-maker
- Medically documented allergy towards the adhesive (glue) of plasters or unable to tolerate tape adhesive in the area of sensor placement
- Serious skin diseases (e.g. psoriasis vulgaris, bacterial skin diseases) located at places of the body, which potentially are possible to be used for localisation of the glucose sensor)
- Currently abusing alcohol, illicit drugs or prescription drugs
- Using pramlintide (symlin) or other commonly used hypoglycaemic agents including sulphonylureas, biguanides, DPP4-inhibitors, GLP-1 agonists, SGLT-2 inhibitors at time of screening
- Elective surgery planned that requires general anaesthesia during the course of the study
- Shift worker with working hours between 10pm and 8am
- Sickle cell disease or haemoglobinopathy
- Red blood cell transfusion or erythropoietin within 3 months prior to time of screening or plans to receive red blood cell transfusion or erythropoietin over the course of study participation

For peer review only

- Diagnosed with current eating disorder such as anorexia or bulimia
- Plans to use significant quantity of herbal preparations (use of over the counter herbal preparation for 30 consecutive days or longer period during the study) or significant quantity of vitamin supplements (four times the recommended daily allowance used for 30 consecutive days or longer period during the study) which may affect glucose metabolism and/or blood glucose levels during the course of their participation in the study

Study schedule

The study will consist of up to 11 in-clinic visits and 6 pre-planned telephone/email contacts. Overview and key activities of the study visits are shown in Table 2 and Table 3, with both intervention periods lasting 3 months.

Study training

Following written informed consent, participants will be trained on the use of study insulin pump (modified Medtronic 640G pump, Medtronic MiniMed, Northridge, CA, USA) and CGM sensor (Enlite 3, Medtronic). For minors, parents/guardians will provide written informed consent, and written assent will be obtained from minors. Training will be performed in the presence of parents/guardians. The study insulin pump will be pre-programmed using participants' usual basal settings, carbohydrate-to-insulin ratio and insulin sensitivity factor. Competency on use of study devices with the participant (co-signature of guardian will be required in USA participants) will be formally assessed.

Run-in period

There will be a minimum 4 weeks run-in period. Participants will be contacted once weekly by phone or email for trouble-shooting purposes. At the end of the run-in period, study devices will be downloaded to assess adherence during last 14 days (participants will be required to demonstrate at least 12 days use of CGM and use of at least 75% of bolus calculator for meal boluses for randomisation). Participants need to perform weekly downloads of study devices.

Randomisation

Eligible subjects will be randomised using central randomisation software to the use of day-and-night closed-loop combined with pump suspend feature or to sensor augmented insulin pump therapy. The randomisation will be stratified at each centre by the two age groups [equal proportion of youth (6 to 21 years) and adults (22 years and older)] and HbA1c groups [equal proportion of below and above 8.5% (69mmol/mol)].

A subset of participants randomised to closed-loop will be invited to take part in an interview study led by an independent team of qualitative researchers at the University of Edinburgh. Participants will be interviewed before and/or at the end of the closed-loop study period.

Home treatment period

All participants will be admitted to a clinical facility at the start of each treatment period and have blood tests performed for HbA1c. Females of child bearing age will have urine pregnancy test on arrival.

For peer review only

Day and night closed-loop combined with pump suspend feature

Participants will be trained on connection and disconnection of the closed-loop system and switching between closed-loop and usual pump therapy. Participants will be instructed on the use of low glucose suspend functionality and settings. During closed-loop period, meal bolus will be delivered by the insulin pump based on carbohydrate estimation. Specific instructions during closed-loop related to exercise management, sick day rules, hypo- and hyperglycaemia management and technical trouble shooting will also be reviewed with the participants by the study team. Competency on the safe and effective use of the closed-loop system will be assessed by the study team, only those who demonstrate the same will be allowed to continue to the home study phase.

Sensor-augmented pump therapy

Participants randomised to sensor-augmented pump therapy will receive training on effective use of real-time CGM for optimisation of insulin therapy. During sensor-augmented pump therapy participants will enter information related to carbohydrate content of the meals and pre-meal capillary glucose values into the bolus wizard of the insulin pump. Participants will be instructed not to activate threshold suspend (low glucose or predictive low glucose suspend features). Hypoglycaemia and hyperglycaemia alarms can be activated and personalised as per participants' requirements.

Contact during 3-month home study period

Participants will have identical planned contact visits with the study team during the two study periods. The first planned contact will occur 24-48 hours once study period has begun. During the first 2 weeks of the study period, participants will be contacted weekly. Thereafter, participants will be contacted monthly. Participants will be free to optimise their treatment independently.

Devices download

Participants will be instructed to download insulin delivery and glucose data weekly from the insulin pump and blood glucose meter using the Medtronic Carelink Clinical download facility.

Closed-loop system and low glucose suspend feature

The FlorenceM closed-loop system (Figure 2, University of Cambridge, Cambridge, UK) comprises a model predictive control algorithm residing on an Android smartphone which communicates wirelessly with the insulin pump using a proprietary enclosure.

Every ten minutes the system calculates a new temporary basal insulin infusion rate which is automatically sent to the study insulin pump. Calculations utilise a compartment model of glucose kinetics describing the effect of rapid-acting insulin analogues and the carbohydrate content of meals on glucose levels[20]. The control algorithm will be initialised using pre-programmed basal insulin delivery downloaded from the study pump. Additionally, information about participants' weight and total daily insulin dose will be entered at set up. During closed-loop operation the algorithm adapts itself to a particular participant. The treat-to-target control algorithm aims to achieve a default glucose level of around 5.8mmol/l and adjusts the actual level depending on fasting versus postprandial status and the accuracy of model-based glucose predictions. Sensor glucose, insulin delivery, carbohydrate content and other information can be visualised by the participants on the smartphone device.

For peer review only

No remote monitoring is instigated. For data collection purposes, the control algorithm device uploads data on a server when the system is charging and connected to Wi-Fi.

The pump comprises CGM receiver and provides hypoglycaemia and hyperglycaemia alarms. Hypoglycaemia and hyperglycaemia alarms can be activated and personalised as per participants' requirements. The insulin pump provides standard insulin pump alarms, and the smartphone alerts the user about aspects related to closed-loop operations (closed-loop started, stopped or terminated).

Safety precautions during closed-loop

Participants will be trained to perform calibration checks before breakfast and evening meals. If sensor glucose is above finger-stick glucose by more than 3.0mmol/l, the glucose sensor will be recalibrated. These instructions resulted from an in-silico simulation of hypo- and hyperglycaemia risk using the validated Cambridge simulator[21].

If sensor glucose becomes unavailable, pre-programmed insulin delivery will be automatically started within 30min. This limits the risk of insulin under- and over-delivery. Safety rules limit maximum insulin infusion and suspend insulin delivery at sensor glucose at or less than 4.3mmol/l or when sensor glucose is rapidly decreasing.

In the event of closed-loop becoming unavailable due to loss of sensor glucose input or other system failures, insulin delivery will be suspended by the low glucose suspend feature set on the insulin pump, provided sensor glucose is available. Resumption of insulin delivery will be in accordance of the low glucose suspend feature implemented on 640G pump.

Participant withdrawal criteria

The following pre-randomisation withdrawal criteria will apply:

1. Participant is unable to demonstrate safe use of study insulin pump and / or CGM during run-in period as judged by the investigator
2. Participant fails to demonstrate compliance with study insulin pump and / or CGM during run-in period

The following pre- and post-randomisation withdrawal criteria will apply:

3. Participants may terminate participation in the study at any time without necessarily giving a reason and without any personal disadvantage
4. Significant protocol violation or non-compliance
5. Any severe hypoglycaemia event related to use of the closed-loop system
6. Three severe hypoglycaemia events unrelated to the use of the closed-loop system
7. Diabetic ketoacidosis (DKA) unrelated to infusion site failure and related to the use of the closed-loop system
8. Decision by the investigator or the sponsor that termination is in the participant's best medical interest
9. Participant becomes pregnant during the study period
10. Allergic reaction to insulin
11. Allergic reaction to adhesive surface of infusion set or CGM sensor
12. If patient continues to use pump suspend feature in the control group despite advice to the contrary

Withdrawn participants due to reasons 4-12 will be invited to provide blood sample at the end of the planned study intervention for the assessment of HbA1c.

For peer review only

Psychosocial methods

A mixed methods psycho-social evaluation using in-depth interviews and validated questionnaires, will be conducted to the determine utility of the device in terms of acceptability of intervention, quality of life, participants' perception of impact on lifestyle and diabetes self-management.

Participants will be invited to complete the PedsQoL questionnaire at screening and at the end of the study intervention. Additionally, a feedback questionnaire on closed-loop specific experience will be distributed to participants/guardians who had been randomised to the closed-loop intervention arm.

A subset of participants (individuals aged 16+ years; individuals aged 13-15 years and their parent/guardian(s); parent/guardian(s) of participants aged 12 years and under) will be invited to take part in an interview at baseline (post-randomisation) to enable their historical diabetes management practices, everyday work/school and family lives, and their initial understandings and expectations of using closed-loop technology to be captured and explored in depth. The same participants will be followed up at the end of the study intervention to determine whether use of the closed-loop matched expectations, whether any difficulties occurred, the benefits and downsides of using closed-loop to support diabetes self-management for the duration of the trial; and, their views about how the technology might be improved.

A subset of partners/family members will be interviewed at 3 months to capture the benefits using a closed-loop system from their perspectives, how this technology has impacted on their own lives and on their role in supporting diabetes management practices.

Recruitment to the interview study will continue until data saturation is achieved; that is, until no new findings are identified in new data collected. It is estimated that around 20-25 adults and youth with diabetes and 20 members will be interviewed. If necessary, participants will be purposely sampled to allow for diversity in the final sample in terms of age, gender, occupation/education and geographical location. Interviews will be digitally recorded with consent.

HbA1c samples

Blood samples for the measurement of HbA1c levels will be taken at 3 different time points. At screening HbA1c will be measured locally. HbA1c at the beginning and end of study intervention will be taken and measured at a central laboratory (University of Minnesota, Minneapolis, USA) using an International Federation of Clinical Chemistry and Laboratory Medicine (IFCC) aligned method.

Statistical analysis

All analysis will be conducted on an intention to treat basis. Data from all randomised participants with or without protocol violation including dropouts and withdrawals will be included in the analysis. Data will not be truncated due to protocol deviations.

Primary endpoint

For peer review only

The primary analysis will evaluate between group difference in the time (midnight to midnight) spent in the target glucose range from 3.9 to 10mmol/l (70 to 180mg/dl) based on sensor glucose levels during the 12-week free living phase.

A 5% significance level will be used to declare statistical significance for the primary outcome comparison.

Mean (SD) for % time spent in the target range will be tabulated by treatment group. A linear model will be used to compare the difference between the two intervention arms, while adjusting for baseline % time spent in the target glucose range and random site effect. A 95% confidence interval will be reported for the difference between the treatment groups based on the linear model. Normality of the residuals will be assessed. If the residuals have highly skewed distribution, then ranked normal score transformation of outcome data will be applied in the regression model. However, previous experience suggests that % time in target glucose range will follow an approximately normal distribution. A detailed analysis plan will be provided separately

Secondary endpoints

The following outcomes on the two treatment arms will be compared:

- HbA1c at 12 weeks
- Time spent below target glucose (3.9mmol/l) (70mg/dl)
- Time spent above target glucose (10.0mmol/l) (180mg/dl)
- Average, standard deviation, and coefficient of variation of glucose levels
- The time with glucose levels < 3.5mmol/l (63mg/dl) and <2.8mmol/l (50mg/dl)
- The time with glucose levels in the significant hyperglycaemia (glucose levels >16.7mmol/l) (300mg/dl)
- Total, basal and bolus insulin dose (units/kg of body weight)
- Area under the curve of glucose below 3.5mmol/l (63mg/dl)
- Number of pump suspend events (applicable to intervention arm)
- Change in body weight from screening to end of study

Glycaemic metrics will be based on sensor glucose levels collected during the 12-week intervention period. Similar linear models as the primary outcome will be used to compare the between treatment difference. For those metrics which have highly skewed distribution, a ranked normal score transformation of outcome data will be applied in the regression model. P-value <0.05 will be used to declare statistical significance for selected secondary outcomes (HbA1c, glucose CV, %time below 3.9mmol/l, %time above 10.0mmol/l, total daily insulin and change in body weight). For other outcomes, to reduce the inflation of type I error caused by multiple comparisons, p-value <0.01 will be used to define statistical significance.

A subset of sensor glucose and insulin metrics will also be tabulated separately for daytime period (8AM-12PM) and night-time period (12PM-8AM).

- Percent time with glucose in target range of 3.9 to 10.0mmol/l (70-180mg/dl)
- Mean glucose
- Glucose variability as measured by standard deviation
- Percent time with glucose level <3.5mmol/l (63mg/dl)
- Amount of delivered insulin

For peer review only

Trends in sensor glucose data collected within intervention arms will be evaluated on a 4-weekly basis.

Safety analysis

The following safety outcomes will be tabulated by treatment group:

- Number of subjects with any diabetic ketoacidosis events
- Number of episodes of diabetic ketoacidosis events per subject and incidence rate per 100-person years
- Number of subjects with any severe hypoglycaemia events
- Number of episodes of severe hypoglycaemia events per subject and incidence rate per 100-person years
- Number of subjects with any severe hyperglycaemia events as defined by fingerprick glucose >16.7mmol/l (>300mg/dl) and plasma ketones >0.6mmol/l
- Number of episodes of severe hyperglycaemia events per subject and incidence rate per 100-person years

Above safety data will be tabulated for all subjects in the two intervention periods, including drop-outs and withdrawals, irrespective of whether sensor glucose data are available and irrespective of whether closed-loop was operational. All adverse events will be listed for the entire study duration.

If there are enough observed events to allow formal statistical modelling for above safety outcomes, the following analyses will be conducted. The event rates will be compared using a Poisson regression model adjusting for random site effect. If outliers exist, a robust Poisson regression model will be used instead. Binary variables will be compared using a logistic regression model adjusting for random site effect. Models involve severe hypoglycaemia events will also adjust for severe hypoglycaemia in the previous 6 months before enrolment.

Utility evaluation

The amount of CGM use will be calculated for both intervention arms, and the amount of closed-loop system used will be calculated for the closed-loop treatment arm only over the 12-week intervention and by 4-weekly period. The difference in CGM use between treatment groups will be compared using similar linear model as described for primary outcome.

Subgroup analysis

No subgroups were considered during the power calculations. Interpretation of any subgroup analyses will depend on whether the overall analysis demonstrates a significant treatment group difference. In the absence of such an overall difference and if performed, the following subgroup analyses will be interpreted with caution: (1) age (6 to 12 years, 13 to 21 years, 22 years and older), (2) gender, (3) race-ethnicity, (4) clinical centre, and (5) baseline HbA1c (<8.5% and ≥8.5%).

Psychosocial evaluation

Questionnaires for diabetes-related quality of life assessment (PedsQL v3.2) will be collected at screening and at the end of the study intervention. For subjects ≥18 years of age, only

For peer review only

answers from the participants themselves will be collected; for subjects ≤17 years of age, answers will be collected from both parent version and child version.

At each assessment time point, mean ± SD score for each dimension and the total score will be tabulated by intervention arm for both parent version and participant version. The between group difference of each score at end of study will be assessed using similar linear model as described previously by adjusting for corresponding score at baseline.

Interview data will be analysed thematically and longitudinally using the method of constant comparison[22]. To maximise rigor at least two experienced qualitative researchers will be involved in the analysis. NVivo9, a qualitative software package, will be used to facilitate data coding/retrieval.

Per-protocol analysis

Per protocol analysis will be conducted to compare the primary endpoint limited to participants who used closed-loop for at least 80% of the of the 3-month study period (with CGM data is available for at least 50% of the study period). In the sensor augmented pump group, per protocol analysis will be conducted on those whose CGM data is available for at least 50% of study period.

Interim analysis

No interim analysis will be performed.

Power calculation

Based on our previous day-and-night closed-loop studies[10,23] and a conservative estimate of 10 percentage points improvement in time when glucose is in target glucose range with a SD of 14.5 percentage points, 76 subjects are required to achieve 85% power and an alpha level of 0.05 (two-tailed). 84 are planned to be randomised to allow for dropouts. It is expected that 95 or more subjects will be recruited; recruitment will continue until it is anticipated that a sufficient number have been enrolled to meet the randomisation goal. Randomisation target may be exceeded since all of the subjects who have initiated the run-in phase will be permitted to continue into the intervention.

STUDY MANAGEMENT

Data Safety Monitoring Board

An independent Data Safety Monitoring Board (DSMB) will comprise a chairperson and two experts. The DSMB will be informed of all SAEs and any unanticipated device effects/events that occur during the study. The DSMB will review compiled adverse event data and periodic intervals. The DSMB will report to the study management committee any safety concerns and recommendations for suspension or early termination of the investigation. Composition of the DSMB is shown in online supplementary appendix.

Study sponsors

In the UK the study sponsor is the Cambridge University Hospitals NHS Foundation Trust, jointly with the University of Cambridge. Study sponsor in the USA is the Jaeb Center for Health Research.

For peer review only

Study management committee

The study management committee consisting of the Chief Investigator, Study Coordinators, and Study Data Manager will confer at least quarterly to discuss the operational aspects of the study. The principal clinical investigators may also participate. Composition of the study management committee is shown in online supplementary appendix.

Study monitoring

The Study Coordinators will ensure that the study is conducted in accordance with Good Clinical Practice standards through site monitoring visits. A monitoring plan will be written and agreed prior to randomisation.

Data management

Confidentiality of participant data shall be observed at all times during the study. Personal details for each participant taking part in the research study and linking them to a unique identification number will be held locally on a study screen log in the Trial Master File at each of the investigation centres. These details will not be revealed at any other stage during the study, and all results will remain anonymous. Study identification number and barcodes will be used on the electronic case report forms and all the blood samples that are collected throughout the study.

Electronic data will be stored on password-protected computers. All paper records will be kept in locked filing cabinets, in a secure office at each of the investigation centres. Only members of the research team and collaborating institutions will have password access to the anonymised electronic data. Only members of the research teams will have access to the filing cabinet. Paper copies of the data will be stored for 15 years.

Direct access to the source data will be provided for monitoring, audits, Research Ethics Committee (REC)/Institutional Review Board (IRB) review and regulatory authority inspections during and after the study. The fully anonymised data may be shared with third parties (EU or non-EU based) for the purposes of advancing management and treatment of diabetes.

Indemnity

Indemnity for any harm rising on the conduct of research will be provided according to local arrangements in respective countries

1) UK- any liability arising from study design will be covered by the clinical trial insurance policy organised by the University of Cambridge. National Health Service indemnity cover will apply for any claims arising from management and conduct of research.

2) USA – any liability arising from study design will be under the responsibility of the participants or their insurance company.

ETHICS AND DISSEMINATION

The study has received approval from independent REC and IRB in the UK and the USA. The study has undergone a review by regulatory authorities in the UK (Medicines and Healthcare products Regulatory Agency) and in the US (Food and Drug Administration). All

For peer review only

participants will be provided with oral and written information about the trial and procedures involved in the study before obtaining written informed consent.

Study operating procedures for monitoring and reporting of all adverse events and adverse device effects will be in place including serious adverse events, serious adverse device effects and specific adverse events such as severe hypoglycaemia and significant hyperglycaemia with ketosis. The DSMB will be informed of all serious adverse events and any unanticipated adverse device/method effects that occur during the study and will review compiled adverse events data at periodic intervals.

Any substantial amendments to the protocol and other documents shall be notified to, and approved by, the independent REC and IRB, and the regulatory authorities, prior to implementation as per nationally agreed guidelines.

It is expected that screening and recruitment will commence in June 2016 and the study will be completed by end of 2017. The study results will be disseminated by peer-review publications and conference presentations.

For peer review only

Acknowledgements: Jasdip Mangat supported development and validation of closed-loop system. Josephine Hayes (Institute of Metabolic Science, University of Cambridge) provided administrative support.

Contributors: RH, HT, MT, DBD, MLE, LL, AC, RMB, and VNS co-designed the study. JL was responsible designing the interview study. RH designed and implemented the glucose controller. LB, HT and RH wrote the manuscript. All authors critically reviewed the report. No writing assistance was provided.

Funding: JDRF, National Institute for Health Research Cambridge Biomedical Research Centre, Wellcome Strategic Award (100574/Z/12/Z)

Competing interest: SH serves as a consultant for Novo-Nordisk and for the ONSET group, and reports having received speaker/training honoraria from Medtronic. MLE reports having received speaker honoraria from Abbott Diabetes Care, Novo Nordisk and Animas, serving on advisory panels for Novo Nordisk, Abbott Diabetes Care, Medtronic, Roche and Cellnovo. RH reports having received speaker honoraria from Eli Lilly, Novo Nordisk and Astra Zeneca, serving on advisory panel for Eli Lilly and Novo Nordisk, receiving license fees from BBraun and Medtronic; and having served as a consultant to BBraun. MEW has received license fees from Becton Dickinson and has served as a consultant to Beckton Dickinson. MT reports having received speaker honoraria from Novo Nordisk and Medtronic. RH and MEW report patent patents and patent applications. LL reports having received speaker honoraria from Minimed Medtronic, Animas, Sanofi and Novo Nordisk, serving on advisory panel for Animas Minimed Medtronic and Novo Nordisk. VNS had received speaking fees from the Dexcom Inc. VNS' employer receives research funding from T1D Exchange and Sanofi. LB, HT, JMA, JE, VH, JS, SB, PC, MB, JL, DE, CLA, FC, AC, MLE, DBD, CK, and RMB declare no competing financial interests exist.

Ethics and IRB approvals: East of England - Cambridge East Research Ethics Committee (UK), Jaeb Center for Health Research Institutional Review (IRB) Office (813-975-8690 or irb@jaeb.org) (USA)

Provenance and peer review: Not commissioned, internally peer reviewed

For peer review only

Table 1. Primary outcome of four randomised free-living unsupervised home day-and-night closed-loop studies.

Study	Duration of home closed-loop (days)	Population	Time spent in target (%)		P value	Reference
			Closed-loop	Control		
Dan04 Phase 1	7	Adolescents	72	53	<0.001	[24]
Dan04 Phase 2	21	Adolescents	67	48	< 0.001	[25]
AP@home02	7	Adults	75	63	0.006	[23]
AP@home04 Phase 1	84	Adults	68	57	<0.001	[10]
AP@home04 Phase 2	28	Adults	76	66	<0.001	[26]

For peer review only

Table 2. Schedule of study visits / phone contacts when the participant is randomised to day and night closed-loop combined with pump suspend feature (intervention group).

	Visit/ contact	Description	Start relative to previous / next Visit / Activity	Duration
	Visit 1	Recruitment visit: Consent, HbA1c, screening bloods & questionnaires, urine pregnancy test	-	1-4 hours
Training & Run-in (4 wks)	Visit 2	Insulin pump training and the initiation study pump Competency assessment	Within 1 to 3 weeks of Visit 1	3-4 hours
	Visit 3	CGM training Initiation of CGM Weekly contact via phone/email Competency assessment	Within 3 to 7 days of Visit 2	2-3 hours
	*Visit 4	Review pump and CGM data & optimisation of treatment & compliance assessment & randomisation	After 4 weeks of Visit 3	<1 hour
	Contact	Qualitative interview (with a subset of participants/family members)	After randomisation but before Visit 5	<1 hour
CL + LGS Intervention (3 months)	Visit 5	CL initiation at clinic/home<br>- urine pregnancy test<br>- CL and suspend feature training<br>- competency assessment<br>- HbA1c	Within 1 week of Visit 4	2-6 hours
	* Visit 6	Review use of study devices	After 24-48 hours of Visit 5	<1 hour
	**Visit 7	Review use of study devices	After 1 week of Visit 5	<1 hour
	*Visit 8	Review pump and CGM data	After 1 week of Visit 7	<1 hour
	*Visit 9	End of first month: Review pump and CGM data	After 2 weeks of Visit 8	<1 hour
	*Visit 10	End of second month: Review pump and CGM data	After 4 weeks of Visit 9	<1 hour
	Visit 11	End of closed-loop treatment arm (3 months): HbA1c, Complete questionnaires, and follow-up qualitative interviews	After 4 weeks of Visit 10	1-3 hours

*Could be done via phone / e-mail in UK. Follow-up by phone is mandatory in USA only.
** Could be done via phone / e-mail in UK. In-person visit mandatory in USA only.

Table 3. Schedule of study visits / phone contacts when the participant is randomised to sensor augmented pump.

	Visit/ contact	Description	Start relative to previous / next Visit / Activity	Duration
	Visit 1	Recruitment visit: Consent HbA1c, screening bloods & questionnaires,<br>urine pregnancy test	-	1-4 hours
**Training & Run-in (4 wks)**	Visit 2	Insulin pump training and the initiation study pump<br>Competency assessment	Within 1 to 3 weeks of Visit 1	3-4 hours
	Visit 3	CGM training<br>Initiation of CGM<br>Weekly contact via phone/email<br>Competency assessment	Within 3 to 7 days of Visit 2	2-3 hours
	*Visit 4	Review pump and CGM data & optimisation of treatment & compliance assessment & randomisation	After 4 weeks of Visit 3	<1 hour
**SAP Intervention (3 months)**	Visit 5	SAP initiation at clinic/home<br>- urine pregnancy test<br>- SAP training<br>- competency assessment<br>- HbA1c	Within 1 week of Visit 4	2-6 hours
	*Visit 6	Review use of study devices	After 24-48 hours of Visit 5	<1 hour
	**Visit 7	Review use of study devices	After 1 week of Visit 5	<1 hour
	*Visit 8	Review pump and CGM data	After 1 week of Visit 7	<1 hour
	*Visit 9	End of first month: Review pump and CGM data	After 2 weeks of Visit 8	<1 hour
	*Visit 10	End of second month: Review pump and CGM data	After 4 weeks of Visit 9	<1 hour
	Visit 11	End of SAP treatment arm (3 months): HbA1c, Complete questionnaires.	After 4 weeks of Visit 10	1-3 hours

* Could be done via phone / e-mail in UK. Follow-up by phone is mandatory in USA only.
** Could be done via phone / e-mail in UK. In-person visit mandatory in USA only.

Figure 1. Study flow chart.

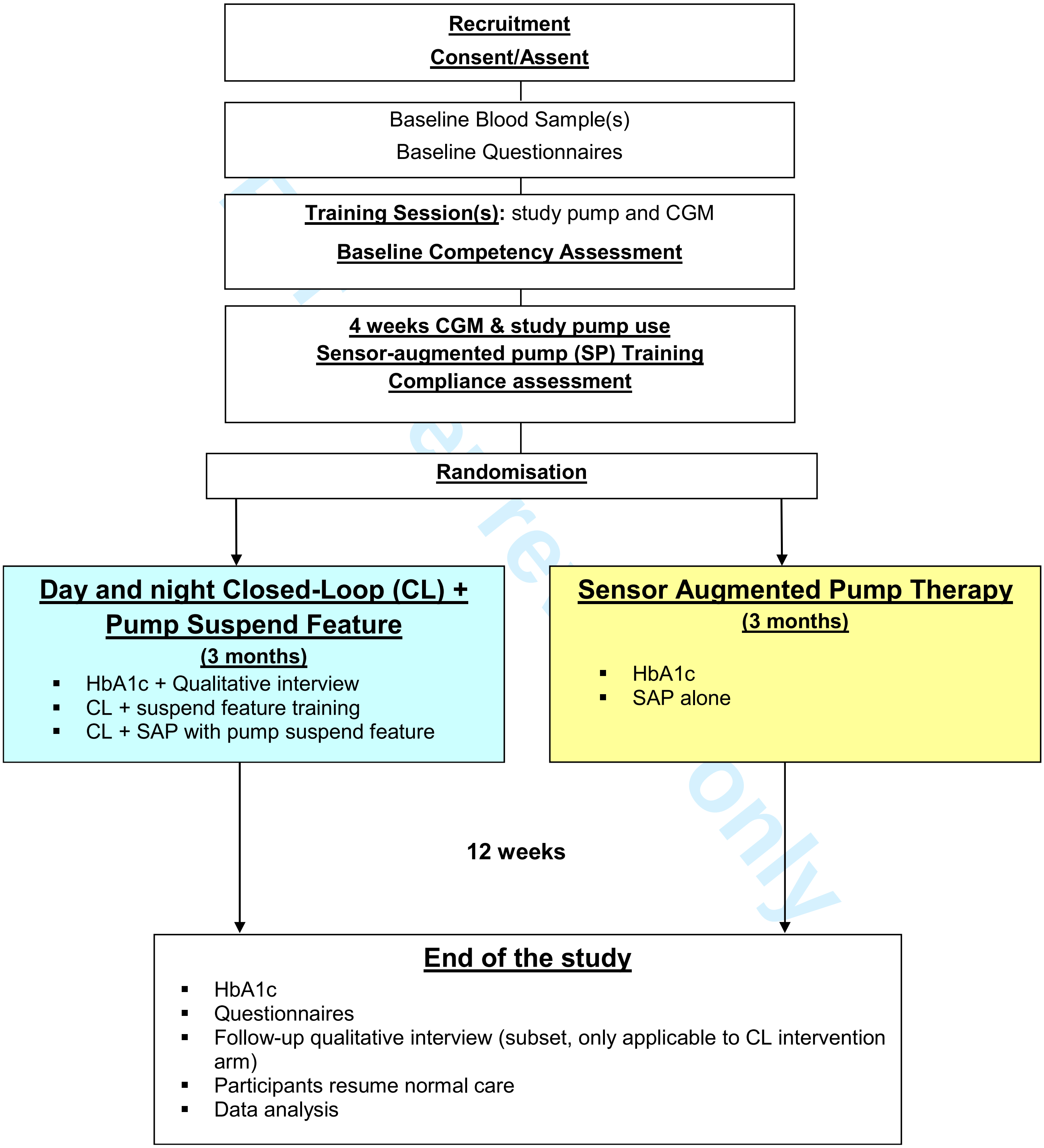

For peer review only

Figure 2. Closed-loop system prototype. (A) Components of the FlorenceM closed-loop system consist of a continuous glucose monitoring (CGM) transmitter with Enlite 3 sensor, an insulin pump (modified 640G pump) integrated with the CGM receiver and a mobile phone running the control algorithm. (B) A photo of a participant (obtained with consent) using the closed-loop system.

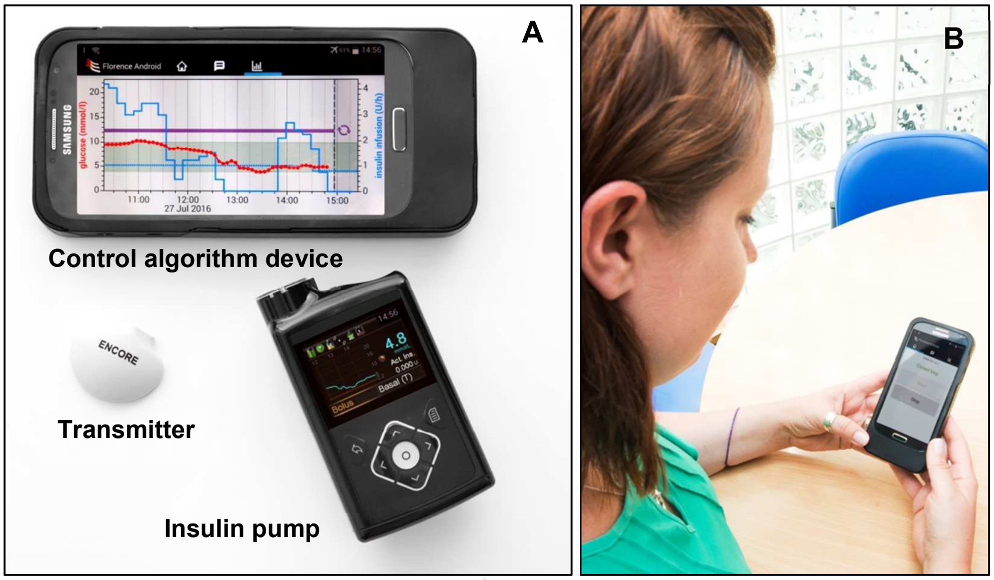

References

1. Atkinson MA, Eisenbarth GS, Michels AW. Type 1 diabetes. *Lancet* 2014; **383**(9911): 69-82.
2. International Diabetes Federation. IDF Diabetes Atlas. 7th Edition 2015. http://www.diabetesatlas.org/ (last accessed 24th March 2016). 2015.
3. Home PD. The pharmacokinetics and pharmacodynamics of rapid-acting insulin analogues and their clinical consequences. *Diabetes Obes Metab* 2012; **14**(9): 780-8.
4. Tamborlane WV, Beck RW, Bode BW, et al. Continuous glucose monitoring and intensive treatment of type 1 diabetes. *N Engl J Med* 2008; **359**(14): 1464-76.
5. Kropff J, DeVries JH. Continuous glucose monitoring, future products, and update on worldwide artificial pancreas projects. *Diabetes Technol Ther* 2016; **18 Suppl 2**: S253-63.
6. Pickup JC, Freeman SC, Sutton AJ. Glycaemic control in type 1 diabetes during real time continuous glucose monitoring compared with self monitoring of blood glucose: meta-analysis of randomised controlled trials using individual patient data. *BMJ* 2011; **343**: d3805.
7. Borges U, Jr., Kubiak T. Continuous glucose monitoring in type 1 diabetes: human factors and usage. *J Diabetes Sci Technol* 2016; **doi: 10.1177/1932296816634736**.
8. Bergenstal RM, Klonoff DC, Garg SK, et al. Threshold-based insulin-pump interruption for reduction of hypoglycemia. *N Engl J Med* 2013; **369**(3): 224-32.
9. Thabit H, Hovorka R. Coming of age: the artificial pancreas for type 1 diabetes. *Diabetologia* 2016; **59**(9): 1795-805.
10. Thabit H, Tauschmann M, Allen JM, et al. Home use of an artificial beta cell in type 1 diabetes. *N Engl J Med* 2015; **373**(22): 2129-40.
11. Kropff J, Del Favero S, Place J, et al. 2 month evening and night closed-loop glucose control in patients with type 1 diabetes under free-living conditions: a randomised crossover trial. *Lancet Diabetes Endocrinol* 2015; **3**(12): 939-47.
12. Bergenstal RM, Garg S, Weinzimer SA, et al. Safety of a hybrid closed-loop insulin delivery system in patients with type 1 diabetes. *JAMA* 2016; **316**(13): 1407-8.
13. Ruan Y, Thabit H, Leelarathna L, et al. Variability of insulin requirements over 12 weeks of closed-loop insulin delivery in adults with type 1 diabetes. *Diabetes Care* 2016; **39**(5): 830-2.
14. Kovatchev B, Cobelli C, Renard E, et al. Multinational study of subcutaneous model-predictive closed-loop control in type 1 diabetes mellitus: summary of the results. *J Diabetes Sci Technol* 2010; **4**(6): 1374-81.
15. Hovorka R, Kumareswaran K, Harris J, et al. Overnight closed loop insulin delivery (artificial pancreas) in adults with type 1 diabetes: crossover randomised controlled studies. *BMJ* 2011; **342**: d1855.
16. Del Favero S, Bruttomesso D, Di Palma F, et al. First use of model predictive control in outpatient wearable artificial pancreas. *Diabetes Care* 2014; **37**(5): 1212-5.
17. Phillip M, Battelino T, Atlas E, et al. Nocturnal glucose control with an artificial pancreas at a diabetes camp. *N Engl J Med* 2013; **368**(9): 824-33.
18. FDA News Release. FDA approves first automated insulin delivery device for type 1 diabetes.http://www.fda.gov/NewsEvents/Newsroom/PressAnnouncements/ucm522974.htm (last accessed 24 October 2016).
19. Seaquist ER, Anderson J, Childs B, et al. Hypoglycemia and diabetes: a report of a workgroup of the American Diabetes Association and the Endocrine Society. *Diabetes Care* 2013; **36**(5): 1384-95.
20. Hovorka R, Shojaee-Moradie F, Carroll PV, et al. Partitioning glucose distribution/transport, disposal, and endogenous production during IVGTT. *Am J Physiol Endocrinol Metab* 2002; **282**(5): E992-1007.
21. Wilinska ME, Chassin LJ, Acerini CL, Allen JM, Dunger DB, Hovorka R. Simulation environment to evaluate closed-loop insulin delivery systems in type 1 diabetes. *J Diabetes Sci Technol* 2010; **4**(1): 132-44.
22. Strauss A, Corbin J. B*asics of qualitative research: grounded theory procedures and techniques. London Sage* 1990.

For peer review only

23. Leelarathna L, Dellweg S, Mader JK, et al. Day and night home closed-loop insulin delivery in adults with type 1 diabetes: three-center randomized crossover study. *Diabetes Care* 2014; **37**(7): 1931-7.
24. Tauschmann M, Allen JM, Wilinska ME, et al. Day-and-night hybrid closed-loop insulin delivery in adolescents with type 1 diabetes: A free-living, randomized clinical trial. *Diabetes Care* 2016; **39**(7): 1168-74.
25. Tauschmann M, Allen JM, Wilinska ME, et al. Home use of day-and-night hybrid closed-loop insulin delivery in suboptimally controlled adolescents with type 1 diabetes: a 3-week, free-living, randomized crossover trial. *Diabetes Care* 2016; **39**(11): 2019-25.
26. Bally L, Thabit H, Kojzar H, et al. Closed-loop insulin delivery in well-controlled type 1 diabetes adults: a free-living day-and-night randomised crossover study. *Lancet Diabetes Endocrinol* 2017. http://dx.doi.org/10.1016/S2213-8587(17)30001-3.

For peer review only

APPENDIX 1

Data Safety Monitoring Board

Dr Irl Hirsch (Chair), University of Washington School of Medicine, Seattle, WA, USA

Dr Michael Tansey (Member), University of Iowa Health Care, IA, USA

Dr Carol Levy (Member), Mount Sinai Hospital, New York, NY, USA

Study Management Team

Chief Investigator

Dr Roman Hovorka, University of Cambridge, Cambridge, UK

Principal Clinical Investigators (Youth)

Dr David Dunger, University of Cambridge, Cambridge, UK

Dr Daniela Elleri, Royal Hospital for Sick Children, Edinburgh, UK

Dr Fiona Campbell, St James's University Hospital, Leeds, UK

Dr Amy Criego, International Diabetes Center, Minneapolis, MN, USA

Principal Clinical Investigators (Adult)

Dr Mark Evans, University of Cambridge, Cambridge, UK

Dr Lalantha Leelarathna, Central Manchester University Hospitals NHS Foundation Trust, Manchester, UK

Dr Richard Bergenstal, International Diabetes Center, Minneapolis, MN, USA

Dr Viral Shah, University of Colorado Denver, Aurora, CO, USA

Clinical Investigators

Dr Carlo L Acerini, University of Cambridge, Cambridge, UK

Dr Hood Thabit, University of Cambridge, Cambridge, UK

Dr Martin Tauschmann, University of Cambridge, Cambridge, UK

Dr Lia Bally, University of Cambridge, Cambridge, UK

Study Co-ordinator (UK)

Mrs Josephine Hayes, University of Cambridge, Cambridge, UK

Study Co-ordinator (USA)

Dr Roy Beck, Jaeb Center for Health Research, Tampa, FL, USA

Medical Sociologist

Dr Julia Lawton, University of Edinburgh, Edinburgh, UK

For peer review only

Study Statistician

Dr Craig Kollman, Jaeb Center for Health Research, Tampa, FL, USA

Study Data Manager

Carlos Murphy, Jaeb Center for Health Research, Tampa, FL, USA

Study Sponsor (UK)

Cambridge University Hospitals NHS Foundation Trust, jointly with University of Cambridge, UK

Study Sponsor (USA)

Jaeb Center for Health Research, Tampa, FL, USA

Funder

JDRF

For peer review only

UNIVERSITY OF CAMBRIDGE

Cambridge University Hospitals NHS
NHS Foundation Trust

IMS Institute of Metabolic Science

Patient ID Label:

Centre NRES number: ....

Patient identification number for this trial: …………………….

For peer review only

CONSENT FORM

(Adults and Youth aged 16 and over)

Title of Project: Home testing of day and night closed loop with pump suspend feature

Name of Researcher: ....

Please initial box

1. I confirm that I have read and understood the information sheet version 2.1 dated 17/11/2015 for the above study and have had the opportunity to consider the information and I am satisfied with the answers I have been given. ☐

2. I understand that my participation is voluntary and that I am free to withdraw at any time, without giving any reason, without my medical care or legal rights being affected in any way.

3. I give consent for the taking of blood and urine samples.

4. If I want to change my mind in the future and withdraw my consent for this study, then I understand that, if I request it, the samples will be destroyed.

5. I give consent to my GP being informed of my participation in this study.

6. I give consent to the sharing of anonymised research data arising from this study with other study partners in Europe and USA.

7. I give consent to anonymised blood samples being sent to USA for analysis.

8. If I choose to communicate with the research team via email, I acknowledge the associated risks as outlined in the information sheet.

9. I understand that relevant sections of my medical notes and data collected during the study may be looked at by individuals from the NHS Trust and regulatory bodies for audit or monitoring purposes.

10. If randomised to the closed loop study arm:

    a. I agree to participate in two qualitative interviews and for my contact details to be given to the study psychologist.

    b. I agree to have my family member/partner contacted by the study psychologist and to have their contact details forwarded.

    c. I agree for my interviews to be digitally recorded.

    d. I understand that anonymised quotes may be used in reports and publications.

11. I agree to return the study devices at the end of the study or earlier if consent is withdrawn.

12. I understand that in the unlikely event that study devices are not returned, legal measures may be undertaken, as a last resort after all other options have been exhausted, to recover the study devices.

13. I agree to take part in the study, and to follow the study team's instructions for the purposes of the study.

For peer review only

14. I give consent for my personal data to be kept by the clinical research team for up to 5 years after termination of the study so that they may contact me in the future with results of this and other similar studies. ☐

Name of participant	Date	Signature
Name of Person taking consent (if different from researcher)	Date	Signature
Researcher	Date	Signature

Copies: *1 for volunteer; 1 for researcher; 1 to be kept with hospital notes*

For peer review only

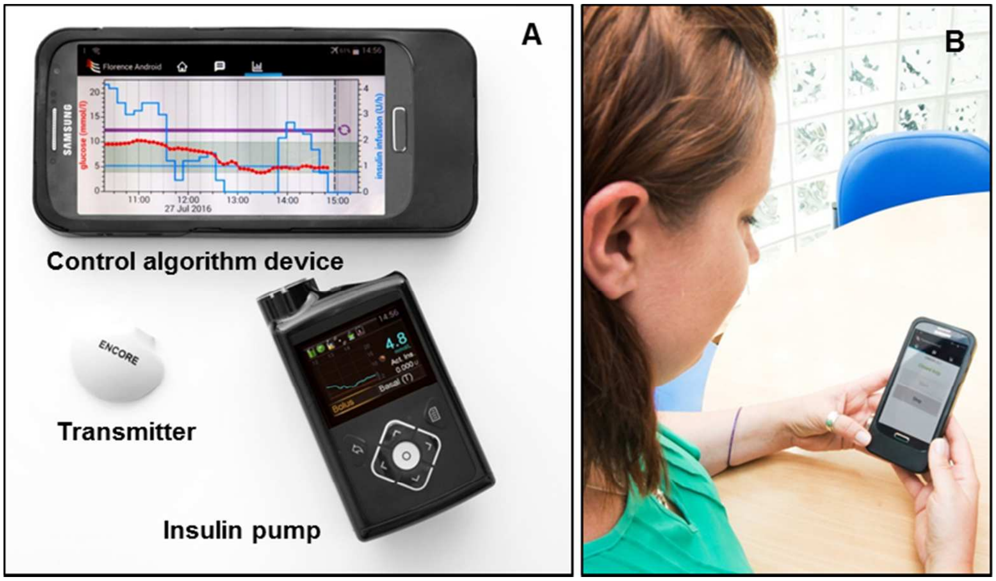

70x40mm (300 x 300 DPI)

For peer review only

STANDARD PROTOCOL ITEMS: RECOMMENDATIONS FOR INTERVENTIONAL TRIALS

SPIRIT 2013 Checklist: Recommended items to address in a clinical trial protocol and related documents*

Section/item	ItemNo	Description	Page number
**Administrative information**			
Title	1	Descriptive title identifying the study design, population, interventions, and, if applicable, trial acronym	1
Trial registration	2a	Trial identifier and registry name. If not yet registered, name of intended registry	2
	2b	All items from the World Health Organization Trial Registration Data Set	
Protocol version	3	Date and version identifier	5
Funding	4	Sources and types of financial, material, and other support	16
Roles and responsibilities	5a	Names, affiliations, and roles of protocol contributors	1, 16
	5b	Name and contact information for the trial sponsor	13
	5c	Role of study sponsor and funders, if any, in study design; collection, management, analysis, and interpretation of data; writing of the report; and the decision to submit the report for publication, including whether they will have ultimate authority over any of these activities	16
	5d	Composition, roles, and responsibilities of the coordinating centre, steering committee, endpoint adjudication committee, data management team, and other individuals or groups overseeing the trial, if applicable (see Item 21a for data monitoring committee)	14
**Introduction**			4

For peer review only

Background and rationale	6a	Description of research question and justification for undertaking the trial, including summary of relevant studies (published and unpublished) examining benefits and harms for each intervention	3
	6b	Explanation for choice of comparators	3
Objectives	7	Specific objectives or hypotheses	3
Trial design	8	Description of trial design including type of trial (eg, parallel group, crossover, factorial, single group), allocation ratio, and framework (eg, superiority, equivalence, noninferiority, exploratory)	5
**Methods: Participants, interventions, and outcomes**			
Study setting	9	Description of study settings (eg, community clinic, academic hospital) and list of countries where data will be collected. Reference to where list of study sites can be obtained	5, 7, 8
Eligibility criteria	10	Inclusion and exclusion criteria for participants. If applicable, eligibility criteria for study centres and individuals who will perform the interventions (eg, surgeons, psychotherapists)	5, 6
Interventions	11a	Interventions for each group with sufficient detail to allow replication, including how and when they will be administered	8, 9
	11b	Criteria for discontinuing or modifying allocated interventions for a given trial participant (eg, drug dose change in response to harms, participant request, or improving/worsening disease)	9
	11c	Strategies to improve adherence to intervention protocols, and any procedures for monitoring adherence (eg, drug tablet return, laboratory tests)	7, 8
	11d	Relevant concomitant care and interventions that are permitted or prohibited during the trial	8

Outcomes	12	Primary, secondary, and other outcomes, including the specific measurement variable (eg, systolic blood pressure), analysis metric (eg, change from baseline, final value, time to event), method of aggregation (eg, median, proportion), and time point for each outcome. Explanation of the clinical relevance of chosen efficacy and harm outcomes is strongly recommended	11, 12
Participant timeline	13	Time schedule of enrolment, interventions (including any run-ins and washouts), assessments, and visits for participants. A schematic diagram is highly recommended (see Figure)	7, 8, 18, 19, 20
Sample size	14	Estimated number of participants needed to achieve study objectives and how it was determined, including clinical and statistical assumptions supporting any sample size calculations	13
Recruitment	15	Strategies for achieving adequate participant enrolment to reach target sample size	5, 13
**Methods: Assignment of interventions (for controlled trials)**			
Allocation:			
Sequence generation	16a	Method of generating the allocation sequence (eg, computer-generated random numbers), and list of any factors for stratification. To reduce predictability of a random sequence, details of any planned restriction (eg, blocking) should be provided in a separate document that is unavailable to those who enrol participants or assign interventions	7
Allocation concealment mechanism	16b	Mechanism of implementing the allocation sequence (eg, central telephone; sequentially numbered, opaque, sealed envelopes), describing any steps to conceal the sequence until interventions are assigned	7
Implementation	16c	Who will generate the allocation sequence, who will enrol participants, and who will assign participants to interventions	5, 7

Blinding (masking)	17a	Who will be blinded after assignment to interventions (eg, trial participants, care providers, outcome assessors, data analysts), and how	N/A
	17b	If blinded, circumstances under which unblinding is permissible, and procedure for revealing a participant's allocated intervention during the trial	N/A
**Methods: Data collection, management, and analysis**			
Data collection methods	18a	Plans for assessment and collection of outcome, baseline, and other trial data, including any related processes to promote data quality (eg, duplicate measurements, training of assessors) and a description of study instruments (eg, questionnaires, laboratory tests) along with their reliability and validity, if known. Reference to where data collection forms can be found, if not in the protocol	8, 10, 12
	18b	Plans to promote participant retention and complete follow-up, including list of any outcome data to be collected for participants who discontinue or deviate from intervention protocols	9, 12
Data management	19	Plans for data entry, coding, security, and storage, including any related processes to promote data quality (eg, double data entry; range checks for data values). Reference to where details of data management procedures can be found, if not in the protocol	14
Statistical methods	20a	Statistical methods for analysing primary and secondary outcomes. Reference to where other details of the statistical analysis plan can be found, if not in the protocol	10, 11, 12, 13
	20b	Methods for any additional analyses (eg, subgroup and adjusted analyses)	12, 13
	20c	Definition of analysis population relating to protocol non-adherence (eg, as randomised analysis), and any statistical methods to handle missing data (eg, multiple imputation)	12, 13
**Methods: Monitoring**			

Data monitoring	21a	Composition of data monitoring committee (DMC); summary of its role and reporting structure; statement of whether it is independent from the sponsor and competing interests; and reference to where further details about its charter can be found, if not in the protocol. Alternatively, an explanation of why a DMC is not needed	13
	21b	Description of any interim analyses and stopping guidelines, including who will have access to these interim results and make the final decision to terminate the trial	13
Harms	22	Plans for collecting, assessing, reporting, and managing solicited and spontaneously reported adverse events and other unintended effects of trial interventions or trial conduct	12
Auditing	23	Frequency and procedures for auditing trial conduct, if any, and whether the process will be independent from investigators and the sponsor	14
**Ethics and dissemination**			
Research ethics approval	24	Plans for seeking research ethics committee/institutional review board (REC/IRB) approval	14, 15
Protocol amendments	25	Plans for communicating important protocol modifications (eg, changes to eligibility criteria, outcomes, analyses) to relevant parties (eg, investigators, REC/IRBs, trial participants, trial registries, journals, regulators)	15
Consent or assent	26a	Who will obtain informed consent or assent from potential trial participants or authorised surrogates, and how (see Item 32)	5, 7
	26b	Additional consent provisions for collection and use of participant data and biological specimens in ancillary studies, if applicable	N/A
Confidentiality	27	How personal information about potential and enrolled participants will be collected, shared, and maintained in order to protect confidentiality before, during, and after the trial	14

For peer review only

Declaration of interests	28	Financial and other competing interests for principal investigators for the overall trial and each study site	16
Access to data	29	Statement of who will have access to the final trial dataset, and disclosure of contractual agreements that limit such access for investigators	14
Ancillary and post-trial care	30	Provisions, if any, for ancillary and post-trial care, and for compensation to those who suffer harm from trial participation	N/A
Dissemination policy	31a	Plans for investigators and sponsor to communicate trial results to participants, healthcare professionals, the public, and other relevant groups (eg, via publication, reporting in results databases, or other data sharing arrangements), including any publication restrictions	15
	31b	Authorship eligibility guidelines and any intended use of professional writers	N/A
	31c	Plans, if any, for granting public access to the full protocol, participant-level dataset, and statistical code	N/A
**Appendices**			
Informed consent materials	32	Model consent form and other related documentation given to participants and authorised surrogates	3, 4, 5
Biological specimens	33	Plans for collection, laboratory evaluation, and storage of biological specimens for genetic or molecular analysis in the current trial and for future use in ancillary studies, if applicable	N/A

*It is strongly recommended that this checklist be read in conjunction with the SPIRIT 2013 Explanation & Elaboration for important clarification on the items. Amendments to the protocol should be tracked and dated. The SPIRIT checklist is copyrighted by the SPIRIT Group under the Creative Commons "Attribution-NonCommercial-NoDerivs 3.0 Unported" license.

For peer review only

BMJ Open

Assessing the effectiveness of 3 months day-and-night home closed-loop control combined with pump suspend feature compared to sensor augmented pump therapy in youths and adults with sub-optimally controlled type 1 diabetes: a randomised parallel study protocol

Journal:	*BMJ Open*
Manuscript ID	bmjopen-2017-016738.R1
Article Type:	Protocol
Date Submitted by the Author:	27-Apr-2017
Complete List of Authors:	Bally, Lia; University of Cambridge, Wellcome-Trust MRC Institute of Metabolic Science; Cambridge University Hospitals NHS Foundation Trust<br>Thabit, Hood; University of Cambridge, Wellcome Trust-MRC Institute of Metabolic Science; Cambridge University Hospitals NHS Foundation Trust<br>Tauschmann, Martin; University of Cambridge; University of Cambridge, Department of Paediatrics<br>Allen, Janet; University of Cambridge, Wellcome Trust-MRC Institute of Metabolic Science; University of Cambridge, Department of Paediatrics<br>Hartnell, Sara; Cambridge University Hospitals NHS Foundation Trust<br>Wilinska, Malgorzata; University of Cambridge, Wellcome Trust-MRC Institute of Metabolic Science; University of Cambridge, Department of Paediatrics<br>Exall, Jane; Leeds Children's Hospital<br>Huegel, Viki; Jaeb Center for Health Research<br>Sibayan, Judy; Jaeb Center for Health Research<br>Borgman, Sarah; Jaeb Center for Health Research<br>Cheng, Peiyao; Jaeb Center for Health Research<br>Blackburn, Maxine; University of Edinburgh, Centre for Population Health Sciences<br>Lawton, Julia; University of Edinburgh, Centre for Population Health Sciences<br>Elleri, Daniela; Royal Hospital for Sick Children<br>Leelarathna, Lalantha; Central Manchester University Hospitals NHS Foundation Trust, Manchester Academic Health Science Centre, Manchester Diabetes Centre<br>Acerini, Carlo; University of Cambridge, Department of Paediatrics<br>Campbell, Fiona; Leeds Children's Hospital<br>Shah, Viral; University of Colorado, Barbara Davis Center for Diabetes<br>Criego, Amy; International Diabetes Center at Park Nicollet<br>Evans, Mark; University of Cambridge, Wellcome Trust-MRC Institute of Metabolic Science; Cambridge University Hospitals NHS Foundation Trust<br>Dunger, David; University of Cambridge, Department of Paediatrics<br>Kollman, Craig; Jaeb Center for Health Research<br>Bergenstal, Richard; International Diabetes Center at Park Nicollet<br>Hovorka, Roman; University of Cambridge, Wellcome Trust-MRC Institute of Metabolic Science; University of Cambridge, Department of Paediatrics
Primary Subject	Diabetes and endocrinology

Heading:	
Secondary Subject Heading:	Medical management
Keywords:	type 1 diabetes, closed-loop, artificial pancreas

For peer review only

Assessing the effectiveness of 3 months day-and-night home closed-loop control combined with pump suspend feature compared to sensor augmented pump therapy in youths and adults with sub-optimally controlled type 1 diabetes: a randomised parallel study protocol

[1,2]Bally L, [1,2]Thabit H, [1,3]Tauschmann M, [1,3]Allen JM, [2]Hartnell S, [1,3]Wilinska ME, [4]Exall J, [5]Huegel V, [5]Sibayan J, [5]Borgman S, [5]Cheng P, [6]Blackburn M, [6]Lawton J, [7]Elleri D, [8]Leelarathna L, [1,3]Acerini CL, [4]Campbell F, [9]Shah VN, [10]Criego A, [1,2]Evans ML, [1,3]Dunger DB, [5]Kollman C, [10]Bergenstal RM, and [1,3]Hovorka R

[1]Wellcome Trust-MRC Institute of Metabolic Science, University of Cambridge, Cambridge, UK

[2]Department of Diabetes & Endocrinology, Cambridge University Hospitals NHS Foundation Trust, Cambridge, UK

[3]Department of Paediatrics, University of Cambridge, Cambridge, UK

[4]Leeds Children's Hospital, Leeds, UK

[5]Jaeb Center for Health Research, Tampa, FL, USA

[6]Centre for Population Health Sciences, University of Edinburgh, Edinburgh, UK

[7]Royal Hospital for Sick Children, Edinburgh, UK

[8]Manchester Diabetes Centre, Central Manchester University Hospitals NHS Foundation Trust, Manchester Academic Health Science Centre, Manchester, UK

[9]Barbara Davis Center for Diabetes, University of Colorado Denver, Aurora, CO, USA

[10]International Diabetes Center at Park Nicollet, MN, USA

Correspondence

Roman Hovorka, PhD

University of Cambridge Metabolic Research Laboratories and NIHR Cambridge Biomedical Research Centre,

Wellcome Trust-MRC Institute of Metabolic Science, Box 289,

Addenbrooke's Hospital, Hills Road, Cambridge CB2 0QQ, UK

tel: +44 1223 762 862, fax: +44 1223 330 598, e-mail: rh347@cam.ac.uk

Key Words: Type 1 diabetes, Closed-loop, Artificial pancreas

Word count abstract: 287

Word count paper: 4924

For peer review only

ABSTRACT

Introduction Despite therapeutic advances, many individuals with type 1 diabetes are unable to achieve tight glycaemic target without increasing the risk of hypoglycaemia. The objective of the present study is to determine the effectiveness of 3-month day-and-night home closed-loop glucose control combined with a pump suspend feature, compared to sensor-augmented insulin pump therapy in youths and adults with sub-optimally controlled type 1 diabetes.

Methods and analysis The study adopts an open-label, multi-centre, multi-national (UK and USA), randomised, single-period, parallel design and aims for 84 randomised patients. Participants are youths (6-21 years) or adults (>21 years) with type 1 diabetes treated with insulin pump therapy and sub-optimal glycaemic control [glycated haemoglobin (HbA1c) ≥7.5% (58mmol/mol) and ≤10% (86mmol/mol)]. Following a 4-week run-in period, eligible participants will be randomised to 3-month use of automated closed-loop insulin delivery combined with pump suspend feature, or to sensor-augmented insulin pump therapy. Analyses will be conducted on an intention to treat basis. The primary outcome is the time spent in the target glucose range from 3.9 to 10.0mmol/l based on continuous glucose monitoring levels during the 3-month free living phase. Secondary outcomes include HbA1c at 3 months, mean glucose, time spent below and above target; time with glucose levels <3.5mmol/l and <2.8mmol/l; area under the curve when sensor glucose is <3.5mmol/l, time with glucose levels >16.7mmol/l, glucose variability; total, basal and bolus insulin dose, change in body weight. Participants' and their families' perception in terms of lifestyle change, daily diabetes management and fear of hypoglycaemia will be evaluated.

Ethics and dissemination Ethics/institutional review board approval has been obtained. Before screening, all participants/guardians will be provided with oral and written information about the trial. The study will be disseminated by peer-review publications and conference presentations.

Trial registration: NCT02523131 (ClinicalTrials.gov)

For peer review only

Strengths and limitations of the study

Strengths

- The study adopts a multi-centre multi-national randomised parallel design
- The study includes youth and adult participants across wide range age groups and geographical locations
- Participants in both study arms have equal numbers of study visits

Limitations

- The study excludes participants who are hypoglycaemia unaware, living alone and those with HbA1c below 7.5% and above 10%
- The study is open-label and the comparator does not include the use of pump suspend feature

For peer review only

Introduction

Type 1 diabetes mellitus (T1D) is caused by an immune-mediated destruction of insulin-producing pancreatic ß-cells resulting in insulin deficiency[1]. It commonly presents in childhood, but one fourth of cases are diagnosed in adults. T1D accounts for 5-10% of the total diabetes cases worldwide[2]. The incidence of T1D is still increasing by around 3% every year, particularly among children[2]. Around 86,000 children develop T1D each year and in 2015 the number of children with T1D exceeded half a million for the first time[2].

Despite rapid advancements in insulin delivery and the on-going development of more physiological insulin preparations[3], achieving optimal glycaemic control while avoiding hypoglycaemia remains a challenge for many people with T1D. The emergence of continuous glucose monitoring (CGM) over the last decade[4,5], which enables users to view real-time interstitial glucose readings and receive alarms for impending hypo- or hyperglycaemia facilitating appropriate changes in insulin therapy, is a major step towards improved diabetes monitoring. Studies have shown clinical benefit of CGM in reducing HbA1c with regular use of the device[6]. Sensor-augmented pumps (SAP) combine real-time CGM with insulin pump therapy and studies have shown HbA1c reduction compared to multiple daily injection basal-bolus therapy[7]. Automatic suspension of insulin delivery by the pump when a predefined threshold is reached (low glucose suspend feature) has been demonstrated to significantly reduce the burden of hypoglycaemia[8].

The development of a closed-loop system that combines glucose monitoring with computer-based algorithm directed insulin delivery[9] may provide further improvements in glycaemic control while reducing hypoglycaemia[10,11] and represents an emerging treatment option for people with T1D[12]. Closed-loop differs from conventional pump therapy, characterised by pre-programmed basal delivery, through the use of a control algorithm which directs subcutaneous insulin delivery according to sensor glucose levels. People with T1D are known to have significant variability in daily insulin requirements[13], which may be addressed by closed-loop.

Closed-loop studies have progressed from fully supervised research facility settings[14,15], to remotely monitored transitional settings such as diabetes camp and hotel settings[16,17]. The use of closed-loop during unsupervised free living over extended periods represents the ultimate testing bed within the target environment (Table 1). The longest randomised day-and-night home study to date have shown benefit in glycaemic control with concurrent reduction of the risk of hypoglycaemia[10]. Based on a recent non-randomised 3-month pivotal study[12], the US Food and Drug Agency (FDA) approved the insulin-only closed-loop system for use in clinical practice[18].

No study thus far has combined the pump suspend feature[8] with closed-loop insulin delivery. In previous closed-loop systems, pre-programmed basal insulin rates are delivered in the event of communication failure between control algorithm device and the insulin pump. In the present closed-loop system, pump inbuilt pump suspend feature is immune to such communication failures and will operate even if the control algorithm device is not in range reducing the risk of prolonged hypoglycaemia. Thus, the addition of pump suspend feature to a closed-loop system further increases user safety by discontinuing insulin delivery at low sensor glucose levels.

For peer review only - http://bmjopen.bmj.com/site/about/guidelines.xhtml

We plan to assess the efficacy, safety, utility and acceptability of 3-month day-and-night home closed-loop glucose control combined with pump suspend feature in youths and adults under free-living conditions compared to sensor-augmented pump therapy.

Methods and Analysis

Overview

This is an open-label, multi-centre, multi-national, randomised, one-period, parallel study contrasting day-and-night automated closed-loop glucose control combined with pump suspend feature (threshold suspend), to sensor augmented insulin pump therapy during free-living home setting (Figure 1, protocol version 5.0 dated 28 February 2017). Study participants will include youths and adults with sub-optimally controlled T1D treated with insulin pump therapy. Recruited participants will be randomly assigned to 3 months of study intervention. The study will aim to randomise 84 participants stratified at each study centre according to the two age groups, which will be based on the age of minors in the USA [equal proportions of youths (6-21 years), and adults (22 years and older)]. The University of Cambridge (UK) and Jaeb Center (USA) will be the coordinating centres. Clinical sites include:

1) Addenbrooke's Hospital, Cambridge, UK (adults and youths)
2) Royal Hospital for Sick Children, Edinburgh, UK (youths)
3) Leeds Teaching Hospital, Leeds, UK (youths)
4) Manchester Royal Infirmary, Manchester, UK (adults)
5) International Diabetes Center at Park Nicollet, Minneapolis, USA (adults and youths)
6) Barbara Davis Center for Childhood Diabetes, Aurora, USA (adults)

Qualitative interviews will be carried by University of Edinburgh, UK. Written informed consent/assent will be obtained from all participants and guardians, as appropriate, before any study related activities.

Inclusion criteria

- At least 6 years or older [with equal proportion of youths (6 to 21 years) and adults (22 years and older)]
- T1D as defined by WHO for at least 1 year or confirmed C-peptide negative
- An insulin pump user for at least 3 months, with good knowledge of insulin self-adjustment as judged by the investigator
- Treated with one of the U-100 rapid acting insulin analogues only (insulin aspart, lispro but not glulisine)
- Willing to perform regular capillary blood glucose monitoring, with at least 4 blood glucose measurements taken every day
- Screening HbA1c ≥ 7.5% (58.5mmol/mol) and ≤ 10% (86mmol/mol) based on analysis from local laboratory or equivalent
- Literate in English
- Willing to wear CGM
- Willing to wear closed-loop system at home
- Willing to follow study specific instructions
- Willing to upload pump and CGM data at regular intervals

For peer review only

- Willing to restrict alcohol consumption to ≤ 2 units per day throughout the study period
- Female participants of child bearing age should be on effective contraception and must have a negative urine-HCG pregnancy test at screening
- Living with someone who is trained to administer intramuscular glucagon and is able to seek emergency assistance
- Access to Wi-Fi at home

Exclusion criteria

- Non-type 1 diabetes mellitus including those secondary to chronic disease
- Using real-time CGM on regular basis in preceding 3 months
- Any other physical or psychological disease likely to interfere with the normal conduct of the study and interpretation of the study results as judged by the investigator
- Untreated coeliac disease, adrenal insufficiency, or hypothyroidism
- Current treatment with drugs known to interfere with glucose metabolism, e.g. systemic corticosteroids, non-selective beta-blockers and MAO inhibitors etc.
- Known or suspected allergy to insulin
- Clinically significant nephropathy (estimated glomerular filtration rate< 45ml/min) or on dialysis, neuropathy or active retinopathy (defined as presence of maculopathy or proliferative changes) as judged by the investigator
- Adults: one or more episodes of severe hypoglycaemia as defined by American Diabetes Association[19] in preceding 6 months; youths: one or more episodes of severe hypoglycaemia during the previous 6 months (adults and adolescents: severe hypoglycaemia is defined as an event requiring assistance of another person to actively administer carbohydrates, glucagon, or take other corrective actions including episodes of hypoglycaemia severe enough to cause unconsciousness, seizures or attendance at hospital; children: severe hypoglycaemia is defined as an event associated with a seizure or loss of consciousness)
- Random C-peptide > 100pmol/l with concomitant blood glucose >4mmol/l (72mg/dl)
- Regular use of acetaminophen
- Lack of reliable telephone facility for contact
- Total daily insulin dose ≥ 2IU/kg/day
- Total daily insulin dose < 15IU/day
- Pregnancy, planned pregnancy, or breast feeding
- Severe visual impairment
- Severe hearing impairment
- Significantly reduced hypoglycaemia awareness in participants 18 year and older defined by Gold score >4
- Using implanted internal pace-maker
- Medically documented allergy towards the adhesive (glue) of plasters or unable to tolerate tape adhesive in the area of sensor placement
- Serious skin diseases (e.g. psoriasis vulgaris, bacterial skin diseases) located at places of the body, which potentially are possible to be used for localisation of the glucose sensor)
- Currently abusing alcohol, illicit drugs or prescription drugs

For peer review only

- Using pramlintide (symlin) or other commonly used hypoglycaemic agents including sulphonylureas, biguanides, DPP4-inhibitors, GLP-1 agonists, SGLT-2 inhibitors at time of screening
- Elective surgery planned that requires general anaesthesia during the course of the study
- Shift worker with working hours between 10pm and 8am
- Sickle cell disease or haemoglobinopathy
- Red blood cell transfusion or erythropoietin within 3 months prior to time of screening or plans to receive red blood cell transfusion or erythropoietin over the course of study participation
- Diagnosed with current eating disorder such as anorexia or bulimia
- Plans to use significant quantity of herbal preparations (use of over the counter herbal preparation for 30 consecutive days or longer period during the study) or significant quantity of vitamin supplements (four times the recommended daily allowance used for 30 consecutive days or longer period during the study) which may affect glucose metabolism and/or blood glucose levels during the course of their participation in the study

Study schedule

The study will consist of up to 11 in-clinic visits and 6 pre-planned telephone/email contacts. Overview and key activities of the study visits are shown in Table 2 and Table 3, with both intervention periods lasting 3 months.

Study training

Following written informed consent, participants will be trained on the use of study insulin pump (modified Medtronic 640G pump, Medtronic MiniMed, Northridge, CA, USA) and CGM sensor (Enlite 3, Medtronic). For minors, parents/guardians will provide written informed consent, and written assent will be obtained from minors. Training will be performed in the presence of parents/guardians. The study insulin pump will be pre-programmed using participants' usual basal settings, carbohydrate-to-insulin ratio and insulin sensitivity factor. Competency on use of study devices with the participant (co-signature of guardian will be required in USA participants) will be formally assessed.

Run-in period

There will be a minimum 4 weeks run-in period. Participants will be contacted once weekly by phone or email for trouble-shooting purposes. At the end of the run-in period, study devices will be downloaded to assess adherence during last 14 days (participants will be required to demonstrate at least 12 days use of CGM and use of at least 75% of bolus calculator for meal boluses for randomisation). Participants need to perform weekly downloads of study devices.

Randomisation

Eligible subjects will be randomised using central randomisation software to the use of day-and-night closed-loop combined with pump suspend feature (threshold suspend) or to sensor augmented insulin pump therapy. The randomisation will be stratified at each centre by the two age groups [equal proportion of youth (6 to 21 years) and adults (22 years and older)] and HbA1c groups [equal proportion of below and above 8.5% (69mmol/mol)].

For peer review only

A subset of participants randomised to closed-loop will be invited to take part in an interview study led by an independent team of qualitative researchers at the University of Edinburgh. Participants will be interviewed before and/or at the end of the closed-loop study period.

Home treatment period

All participants will be admitted to a clinical facility at the start of each treatment period and have blood tests performed for HbA1c. Females of child bearing age will have urine pregnancy test on arrival.

Day and night closed-loop combined with pump suspend feature

Participants will be trained on connection and disconnection of the closed-loop system and switching between closed-loop and usual pump therapy. Participants will be instructed on the use of low glucose suspend functionality and settings. During closed-loop period, meal bolus will be delivered by the insulin pump based on carbohydrate estimation. Specific instructions during closed-loop related to exercise management, sick day rules, hypo- and hyperglycaemia management and technical trouble shooting will also be reviewed with the participants by the study team. Competency on the safe and effective use of the closed-loop system will be assessed by the study team, only those who demonstrate the same will be allowed to continue to the home study phase.

Sensor-augmented pump therapy

Participants randomised to sensor-augmented pump therapy will receive training on effective use of real-time CGM for optimisation of insulin therapy. During sensor-augmented pump therapy participants will enter information related to carbohydrate content of the meals and pre-meal capillary glucose values into the bolus wizard of the insulin pump. Participants will be instructed not to activate threshold suspend (low glucose or predictive low glucose suspend features). Hypoglycaemia and hyperglycaemia alarms can be activated and personalised as per participants' requirements.

Contact during 3-month home study period

Participants will have identical planned contact visits with the study team during the two study periods. The first planned contact will occur 24-48 hours once study period has begun. During the first 2 weeks of the study period, participants will be contacted weekly. Thereafter, participants will be contacted monthly. Participants will be free to optimise their treatment independently.

Devices download

Participants will be instructed to download insulin delivery and glucose data weekly from the insulin pump and blood glucose meter using the Medtronic Carelink Clinical download facility.

Closed-loop system and low glucose suspend feature

The FlorenceM closed-loop system (Figure 2, University of Cambridge, Cambridge, UK) comprises a model predictive control algorithm residing on an Android smartphone which communicates wirelessly with the insulin pump using a proprietary enclosure.

Every ten minutes the system calculates a new temporary basal insulin infusion rate which is automatically sent to the study insulin pump. Calculations utilise a compartment model of

For peer review only

glucose kinetics describing the effect of rapid-acting insulin analogues and the carbohydrate content of meals on glucose levels[20]. The control algorithm will be initialised using pre-programmed basal insulin delivery downloaded from the study pump. Additionally, information about participants' weight and total daily insulin dose will be entered at set up. During closed-loop operation the algorithm adapts itself to a particular participant. The treat-to-target control algorithm aims to achieve a default glucose level of around 5.8mmol/l and adjusts the actual level depending on fasting versus postprandial status and the accuracy of model-based glucose predictions. Sensor glucose, insulin delivery, carbohydrate content and other information can be visualised by the participants on the smartphone device.

No remote monitoring is instigated. For data collection purposes, the control algorithm device uploads data on a server when the system is charging and connected to Wi-Fi.

The pump comprises CGM receiver and provides hypoglycaemia and hyperglycaemia alarms. Hypoglycaemia and hyperglycaemia alarms can be activated and personalised as per participants' requirements. The insulin pump provides standard insulin pump alarms, and the smartphone alerts the user about aspects related to closed-loop operations (closed-loop started, stopped or terminated).

Safety precautions during closed-loop

Participants will be trained to perform calibration checks before breakfast and evening meals. If sensor glucose is above finger-stick glucose by more than 3.0mmol/l, the glucose sensor will be recalibrated. These instructions resulted from an in-silico simulation of hypo- and hyperglycaemia risk using the validated Cambridge simulator[21].

If sensor glucose becomes unavailable, pre-programmed insulin delivery will be automatically started within 30min. This limits the risk of insulin under- and over-delivery. Safety rules limit maximum insulin infusion and suspend insulin delivery at sensor glucose at or less than 4.3mmol/l or when sensor glucose is rapidly decreasing.

In the event of closed-loop becoming unavailable due to loss of sensor glucose input or other system failures, insulin delivery will be suspended by the low glucose suspend feature set on the insulin pump, provided sensor glucose is available. Resumption of insulin delivery will be in accordance of the low glucose suspend feature implemented on 640G pump.

Participant withdrawal criteria

The following pre-randomisation withdrawal criteria will apply:

1. Participant is unable to demonstrate safe use of study insulin pump and / or CGM during run-in period as judged by the investigator
2. Participant fails to demonstrate compliance with study insulin pump and / or CGM during run-in period

The following pre- and post-randomisation withdrawal criteria will apply:

3. Participants may terminate participation in the study at any time without necessarily giving a reason and without any personal disadvantage
4. Significant protocol violation or non-compliance
5. Any severe hypoglycaemia event related to use of the closed-loop system
6. Three severe hypoglycaemia events unrelated to the use of the closed-loop system
7. Diabetic ketoacidosis (DKA) unrelated to infusion site failure and related to the use of the closed-loop system

For peer review only

8. Decision by the investigator or the sponsor that termination is in the participant's best medical interest
9. Participant becomes pregnant during the study period
10. Allergic reaction to insulin
11. Allergic reaction to adhesive surface of infusion set or CGM sensor
12. If patient continues to use pump suspend feature in the control group despite advice to the contrary

Withdrawn participants due to reasons 4-12 will be invited to provide blood sample at the end of the planned study intervention for the assessment of HbA1c.

Psychosocial methods

A mixed methods psycho-social evaluation using in-depth interviews and validated questionnaires, will be conducted to the determine utility of the device in terms of acceptability of intervention, quality of life, participants' perception of impact on lifestyle and diabetes self-management.

Participants will be invited to complete the PedsQoL questionnaire at screening and at the end of the study intervention. Additionally, a feedback questionnaire on closed-loop specific experience will be distributed to participants/guardians who had been randomised to the closed-loop intervention arm.

A subset of participants (individuals aged 16+ years; individuals aged 13-15 years and their parent/guardian(s); parent/guardian(s) of participants aged 12 years and under) will be invited to take part in an interview at baseline (post-randomisation) to enable their historical diabetes management practices, everyday work/school and family lives, and their initial understandings and expectations of using closed-loop technology to be captured and explored in depth. The same participants will be followed up at the end of the study intervention to determine whether use of the closed-loop matched expectations, whether any difficulties occurred, the benefits and downsides of using closed-loop to support diabetes self-management for the duration of the trial; and, their views about how the technology might be improved.

A subset of partners/family members will be interviewed at 3 months to capture the benefits using a closed-loop system from their perspectives, how this technology has impacted on their own lives and on their role in supporting diabetes management practices.

Recruitment to the interview study will continue until data saturation is achieved; that is, until no new findings are identified in new data collected. It is estimated that around 20-25 adults and youth with diabetes and 20 members will be interviewed. If necessary, participants will be purposely sampled to allow for diversity in the final sample in terms of age, gender, occupation/education and geographical location. Interviews will be digitally recorded with consent.

HbA1c samples

Blood samples for the measurement of HbA1c levels will be taken at 3 different time points. At screening HbA1c will be measured locally. HbA1c at the beginning and end of study intervention will be taken and measured at a central laboratory (University of Minnesota,

For peer review only

Minneapolis, USA) using an International Federation of Clinical Chemistry and Laboratory Medicine (IFCC) aligned method.

For peer review only

Statistical analysis

All analysis will be conducted on an intention to treat basis. Data from all randomised participants with or without protocol violation including dropouts and withdrawals will be included in the analysis. Data will not be truncated due to protocol deviations.

Primary endpoint

The primary analysis will evaluate between group difference in the time (midnight to midnight) spent in the target glucose range from 3.9 to 10mmol/l (70 to 180mg/dl) based on sensor glucose levels during the 12-week free living phase.

A 5% significance level will be used to declare statistical significance for the primary outcome comparison.

Mean (SD) for % time spent in the target range will be tabulated by treatment group. A linear model will be used to compare the difference between the two intervention arms, while adjusting for baseline % time spent in the target glucose range and random site effect. A 95% confidence interval will be reported for the difference between the treatment groups based on the linear model. Normality of the residuals will be assessed. If the residuals have highly skewed distribution, then ranked normal score transformation of outcome data will be applied in the regression model. However, previous experience suggests that % time in target glucose range will follow an approximately normal distribution. A detailed analysis plan will be provided separately

Secondary endpoints

The following outcomes on the two treatment arms will be compared:

- HbA1c at 12 weeks
- Time spent below target glucose (3.9mmol/l) (70mg/dl)
- Time spent above target glucose (10.0mmol/l) (180mg/dl)
- Average, standard deviation, and coefficient of variation of glucose levels
- The time with glucose levels < 3.5mmol/l (63mg/dl) and <2.8mmol/l (50mg/dl)
- The time with glucose levels in the significant hyperglycaemia (glucose levels >16.7mmol/l) (300mg/dl)
- Total, basal and bolus insulin dose (units/kg of body weight)
- Area under the curve of glucose below 3.5mmol/l (63mg/dl)
- Number of pump suspend events (applicable to intervention arm)
- Change in body weight from screening to end of study

Glycaemic metrics will be based on sensor glucose levels collected during the 12-week intervention period. Similar linear models as the primary outcome will be used to compare the between treatment difference. For those metrics which have highly skewed distribution, a ranked normal score transformation of outcome data will be applied in the regression model. P-value <0.05 will be used to declare statistical significance for selected secondary outcomes (HbA1c, glucose CV, %time below 3.9mmol/l, %time above 10.0mmol/l, total daily insulin and change in body weight). For other outcomes, to reduce the inflation of type I error caused by multiple comparisons, p-value <0.01 will be used to define statistical significance.

For peer review only

A subset of sensor glucose and insulin metrics will also be tabulated separately for daytime period (8AM-12PM) and night-time period (12PM-8AM).

- Percent time with glucose in target range of 3.9 to 10.0mmol/l (70-180mg/dl)
- Mean glucose
- Glucose variability as measured by standard deviation
- Percent time with glucose level <3.5mmol/l (63mg/dl)
- Amount of delivered insulin

Trends in sensor glucose data collected within intervention arms will be evaluated on a 4-weekly basis.

Safety analysis

The following safety outcomes will be tabulated by treatment group:

- Number of subjects with any diabetic ketoacidosis events
- Number of episodes of diabetic ketoacidosis events per subject and incidence rate per 100-person years
- Number of subjects with any severe hypoglycaemia events
- Number of episodes of severe hypoglycaemia events per subject and incidence rate per 100-person years
- Number of subjects with any severe hyperglycaemia events as defined by fingerprick glucose >16.7mmol/l (>300mg/dl) and plasma ketones >0.6mmol/l
- Number of episodes of severe hyperglycaemia events per subject and incidence rate per 100-person years

Above safety data will be tabulated for all subjects in the two intervention periods, including drop-outs and withdrawals, irrespective of whether sensor glucose data are available and irrespective of whether closed-loop was operational. All adverse events will be listed for the entire study duration.

If there are enough observed events to allow formal statistical modelling for above safety outcomes, the following analyses will be conducted. The event rates will be compared using a Poisson regression model adjusting for random site effect. If outliers exist, a robust Poisson regression model will be used instead. Binary variables will be compared using a logistic regression model adjusting for random site effect. Models involve severe hypoglycaemia events will also adjust for severe hypoglycaemia in the previous 6 months before enrolment.

Utility evaluation

The amount of CGM use will be calculated for both intervention arms, and the amount of closed-loop system used will be calculated for the closed-loop treatment arm only over the 12-week intervention and by 4-weekly period. The difference in CGM use between treatment groups will be compared using similar linear model as described for primary outcome.

Subgroup analysis

No subgroups were considered during the power calculations. Interpretation of any subgroup analyses will depend on whether the overall analysis demonstrates a significant treatment group difference. In the absence of such an overall difference and if performed, the following

For peer review only

subgroup analyses will be interpreted with caution: (1) age (6 to 12 years, 13 to 21 years, 22 years and older), (2) gender, (3) race-ethnicity, (4) clinical centre, and (5) baseline HbA1c (<8.5% and ≥8.5%).

Psychosocial evaluation

Questionnaires for diabetes-related quality of life assessment (PedsQL v3.2) will be collected at screening and at the end of the study intervention. For subjects ≥18 years of age, only answers from the participants themselves will be collected; for subjects ≤17 years of age, answers will be collected from both parent version and child version.

At each assessment time point, mean ± SD score for each dimension and the total score will be tabulated by intervention arm for both parent version and participant version. The between group difference of each score at end of study will be assessed using similar linear model as described previously by adjusting for corresponding score at baseline.

Interview data will be analysed thematically and longitudinally using the method of constant comparison[22]. To maximise rigor at least two experienced qualitative researchers will be involved in the analysis. NVivo9, a qualitative software package, will be used to facilitate data coding/retrieval.

Per-protocol analysis

Per protocol analysis will be conducted to compare the primary endpoint limited to participants who used closed-loop for at least 80% of the of the 3-month study period (with CGM data is available for at least 50% of the study period). In the sensor augmented pump group, per protocol analysis will be conducted on those whose CGM data is available for at least 50% of study period.

Interim analysis

No interim analysis will be performed.

Power calculation

Based on our previous day-and-night closed-loop studies[10,23] and a conservative estimate of 10 percentage points improvement in time when glucose is in target glucose range with a SD of 14.5 percentage points, 76 subjects are required to achieve 85% power and an alpha level of 0.05 (two-tailed). 84 are planned to be randomised to allow for dropouts. It is expected that 95 or more subjects will be recruited; recruitment will continue until it is anticipated that a sufficient number have been enrolled to meet the randomisation goal. Randomisation target may be exceeded since all of the subjects who have initiated the run-in phase will be permitted to continue into the intervention.

STUDY MANAGEMENT

Data Safety Monitoring Board

An independent Data Safety Monitoring Board (DSMB) will comprise a chairperson and two experts. The DSMB will be informed of all SAEs and any unanticipated device effects/events that occur during the study. The DSMB will review compiled adverse event data and periodic intervals. The DSMB will report to the study management committee any safety concerns

For peer review only

and recommendations for suspension or early termination of the investigation. Composition of the DSMB is shown in online supplementary appendix.

Study sponsors

In the UK the study sponsor is the Cambridge University Hospitals NHS Foundation Trust, jointly with the University of Cambridge. Study sponsor in the USA is the Jaeb Center for Health Research.

Study management committee

The study management committee consisting of the Chief Investigator, Study Coordinators, and Study Data Manager will confer at least quarterly to discuss the operational aspects of the study. The principal clinical investigators may also participate. Composition of the study management committee is shown in online supplementary appendix.

Study monitoring

The Study Coordinators will ensure that the study is conducted in accordance with Good Clinical Practice standards through site monitoring visits. A monitoring plan will be written and agreed prior to randomisation.

Data management

Confidentiality of participant data shall be observed at all times during the study. Personal details for each participant taking part in the research study and linking them to a unique identification number will be held locally on a study screen log in the Trial Master File at each of the investigation centres. These details will not be revealed at any other stage during the study, and all results will remain anonymous. Study identification number and barcodes will be used on the electronic case report forms and all the blood samples that are collected throughout the study.

Electronic data will be stored on password-protected computers. All paper records will be kept in locked filing cabinets, in a secure office at each of the investigation centres. Only members of the research team and collaborating institutions will have password access to the anonymised electronic data. Only members of the research teams will have access to the filing cabinet. Paper copies of the data will be stored for 15 years.

Direct access to the source data will be provided for monitoring, audits, Research Ethics Committee (REC)/Institutional Review Board (IRB) review and regulatory authority inspections during and after the study. The fully anonymised data may be shared with third parties (EU or non-EU based) for the purposes of advancing management and treatment of diabetes.

Indemnity

Indemnity for any harm rising on the conduct of research will be provided according to local arrangements in respective countries

1) UK- any liability arising from study design will be covered by the clinical trial insurance policy organised by the University of Cambridge. National Health Service indemnity cover will apply for any claims arising from management and conduct of research.

For peer review only

2) USA – any liability arising from study design will be under the responsibility of the participants or their insurance company.

ETHICS AND DISSEMINATION

The study has received approval from independent REC and IRB in the UK and the USA. The study has undergone a review by regulatory authorities in the UK (Medicines and Healthcare products Regulatory Agency) and in the US (Food and Drug Administration). All participants will be provided with oral and written information about the trial and procedures involved in the study before obtaining written informed consent.

Study operating procedures for monitoring and reporting of all adverse events and adverse device effects will be in place including serious adverse events, serious adverse device effects and specific adverse events such as severe hypoglycaemia and significant hyperglycaemia with ketosis. The DSMB will be informed of all serious adverse events and any unanticipated adverse device/method effects that occur during the study and will review compiled adverse events data at periodic intervals.

Any substantial amendments to the protocol and other documents shall be notified to, and approved by, the independent REC and IRB (UK: Cambridge East Research Ethics Committee, #15/EE/0324; USA: Jaeb Center for Health Research Institutional Review Board certified by the Office for Human Research Protections (FWA #00000024)), and the regulatory authorities, prior to implementation as per nationally agreed guidelines.

Screening and recruitment commenced in June 2016 and the study is expected to be completed by end of 2017. The study results will be disseminated by peer-review publications and conference presentations.

For peer review only

Acknowledgements: Jasdip Mangat supported development and validation of closed-loop system. Josephine Hayes (Institute of Metabolic Science, University of Cambridge) provided administrative support.

Contributors: RH, HT, MT, DBD, MLE, LL, AC, RMB, and VNS co-designed the study. JL was responsible designing the interview study. RH designed and implemented the glucose controller. LB, HT and RH wrote the manuscript. All authors critically reviewed the report. No writing assistance was provided.

Funding: JDRF, National Institute for Health Research Cambridge Biomedical Research Centre, Wellcome Strategic Award (100574/Z/12/Z)

Competing interest: SH serves as a consultant for Novo-Nordisk and for the ONSET group, and reports having received speaker/training honoraria from Medtronic. MLE reports having received speaker honoraria from Abbott Diabetes Care, Novo Nordisk and Animas, serving on advisory panels for Novo Nordisk, Abbott Diabetes Care, Medtronic, Roche and Cellnovo. RH reports having received speaker honoraria from Eli Lilly, Novo Nordisk and Astra Zeneca, serving on advisory panel for Eli Lilly and Novo Nordisk, receiving license fees from BBraun and Medtronic; and having served as a consultant to BBraun. MEW has received license fees from Becton Dickinson and has served as a consultant to Beckton Dickinson. MT reports having received speaker honoraria from Novo Nordisk and Medtronic. RH and MEW report patent patents and patent applications. LL reports having received speaker honoraria from Minimed Medtronic, Animas, Sanofi and Novo Nordisk, serving on advisory panel for Animas Minimed Medtronic and Novo Nordisk. VNS had received speaking fees from the Dexcom Inc. VNS' employer receives research funding from T1D Exchange and Sanofi. LB, HT, JMA, JE, VH, JS, SB, PC, MB, JL, DE, CLA, FC, AC, MLE, DBD, CK, and RMB declare no competing financial interests exist.

Ethics and IRB approvals: East of England - Cambridge East Research Ethics Committee (UK), Jaeb Center for Health Research Institutional Review (IRB) Office (813-975-8690 or irb@jaeb.org) (USA)

Provenance and peer review: Not commissioned, internally peer reviewed

For peer review only

Table 1. Primary outcome of four randomised free-living unsupervised home day-and-night closed-loop studies.

Study	Duration of home closed-loop (days)	Population	Time spent in target (%)		P value	Reference
			Closed-loop	Control		
Dan04 Phase 1	7	Adolescents	72	53	<0.001	[24]
Dan04 Phase 2	21	Adolescents	67	48	< 0.001	[25]
AP@home02	7	Adults	75	63	0.006	[23]
AP@home04 Phase 1	84	Adults	68	57	<0.001	[10]
AP@home04 Phase 2	28	Adults	76	66	<0.001	[26]

For peer review only

Table 2. Schedule of study visits / phone contacts when the participant is randomised to day and night closed-loop combined with pump suspend feature (intervention group).

	Visit/ contact	Description	Start relative to previous / next Visit / Activity	Duration
	Visit 1	Recruitment visit: Consent, HbA1c, screening bloods & questionnaires, urine pregnancy test	-	1-4 hours
Training & Run-in (4 wks)	Visit 2	Insulin pump training and the initiation study pump Competency assessment	Within 1 to 3 weeks of Visit 1	3-4 hours
	Visit 3	CGM training Initiation of CGM Weekly contact via phone/email Competency assessment	Within 3 to 7 days of Visit 2	2-3 hours
	*Visit 4	Review pump and CGM data & optimisation of treatment & compliance assessment & randomisation	After 4 weeks of Visit 3	<1 hour
	Contact	Qualitative interview (with a subset of participants/family members)	After randomisation but before Visit 5	<1 hour
CL + LGS Intervention (3 months)	Visit 5	CL initiation at clinic/home<br>- urine pregnancy test<br>- CL and suspend feature training<br>- competency assessment<br>- HbA1c	Within 1 week of Visit 4	2-6 hours
	* Visit 6	Review use of study devices	After 24-48 hours of Visit 5	<1 hour
	**Visit 7	Review use of study devices	After 1 week of Visit 5	<1 hour
	*Visit 8	Review pump and CGM data	After 1 week of Visit 7	<1 hour
	*Visit 9	End of first month: Review pump and CGM data	After 2 weeks of Visit 8	<1 hour
	*Visit 10	End of second month: Review pump and CGM data	After 4 weeks of Visit 9	<1 hour
	Visit 11	End of closed-loop treatment arm (3 months): HbA1c, Complete questionnaires, and follow-up qualitative interviews	After 4 weeks of Visit 10	1-3 hours

*Could be done via phone / e-mail in UK. Follow-up by phone is mandatory in USA only.
** Could be done via phone / e-mail in UK. In-person visit mandatory in USA only.

Table 3. Schedule of study visits / phone contacts when the participant is randomised to sensor augmented pump.

	Visit/ contact	Description	Start relative to previous / next Visit / Activity	Duration
	Visit 1	Recruitment visit: Consent HbA1c, screening bloods & questionnaires, urine pregnancy test	-	1-4 hours
Training & Run-in (4 wks)	Visit 2	Insulin pump training and the initiation study pump Competency assessment	Within 1 to 3 weeks of Visit 1	3-4 hours
	Visit 3	CGM training Initiation of CGM Weekly contact via phone/email Competency assessment	Within 3 to 7 days of Visit 2	2-3 hours
	*Visit 4	Review pump and CGM data & optimisation of treatment & compliance assessment & randomisation	After 4 weeks of Visit 3	<1 hour
SAP Intervention (3 months)	Visit 5	SAP initiation at clinic/home<br>- urine pregnancy test<br>- SAP training<br>- competency assessment<br>- HbA1c	Within 1 week of Visit 4	2-6 hours
	*Visit 6	Review use of study devices	After 24-48 hours of Visit 5	<1 hour
	**Visit 7	Review use of study devices	After 1 week of Visit 5	<1 hour
	*Visit 8	Review pump and CGM data	After 1 week of Visit 7	<1 hour
	*Visit 9	End of first month: Review pump and CGM data	After 2 weeks of Visit 8	<1 hour
	*Visit 10	End of second month: Review pump and CGM data	After 4 weeks of Visit 9	<1 hour
	Visit 11	End of SAP treatment arm (3 months): HbA1c, Complete questionnaires.	After 4 weeks of Visit 10	1-3 hours

* Could be done via phone / e-mail in UK. Follow-up by phone is mandatory in USA only.
** Could be done via phone / e-mail in UK. In-person visit mandatory in USA only.

Figure 1. Study flow chart.

Figure 2. Closed-loop system prototype. (A) Components of the FlorenceM closed-loop system consist of a continuous glucose monitoring (CGM) transmitter with Enlite 3 sensor, an insulin pump (modified 640G pump) integrated with the CGM receiver and a mobile phone running the control algorithm. (B) A photo of a participant (obtained with consent) using the closed-loop system.

For peer review only

References

1. Atkinson MA, Eisenbarth GS, Michels AW. Type 1 diabetes. *Lancet* 2014; **383**(9911): 69-82.
2. International Diabetes Federation. IDF Diabetes Atlas. 7th Edition 2015. http://www.diabetesatlas.org/ (last accessed 24th March 2016). 2015.
3. Home PD. The pharmacokinetics and pharmacodynamics of rapid-acting insulin analogues and their clinical consequences. *Diabetes Obes Metab* 2012; **14**(9): 780-8.
4. Tamborlane WV, Beck RW, Bode BW, et al. Continuous glucose monitoring and intensive treatment of type 1 diabetes. *N Engl J Med* 2008; **359**(14): 1464-76.
5. Kropff J, DeVries JH. Continuous glucose monitoring, future products, and update on worldwide artificial pancreas projects. *Diabetes Technol Ther* 2016; **18 Suppl 2**: S253-63.
6. Pickup JC, Freeman SC, Sutton AJ. Glycaemic control in type 1 diabetes during real time continuous glucose monitoring compared with self monitoring of blood glucose: meta-analysis of randomised controlled trials using individual patient data. *BMJ* 2011; **343**: d3805.
7. Borges U, Jr., Kubiak T. Continuous glucose monitoring in type 1 diabetes: human factors and usage. *J Diabetes Sci Technol* 2016; **doi: 10.1177/1932296816634736**.
8. Bergenstal RM, Klonoff DC, Garg SK, et al. Threshold-based insulin-pump interruption for reduction of hypoglycemia. *N Engl J Med* 2013; **369**(3): 224-32.
9. Thabit H, Hovorka R. Coming of age: the artificial pancreas for type 1 diabetes. *Diabetologia* 2016; **59**(9): 1795-805.
10. Thabit H, Tauschmann M, Allen JM, et al. Home use of an artificial beta cell in type 1 diabetes. *N Engl J Med* 2015; **373**(22): 2129-40.
11. Kropff J, Del Favero S, Place J, et al. 2 month evening and night closed-loop glucose control in patients with type 1 diabetes under free-living conditions: a randomised crossover trial. *Lancet Diabetes Endocrinol* 2015; **3**(12): 939-47.
12. Bergenstal RM, Garg S, Weinzimer SA, et al. Safety of a hybrid closed-loop insulin delivery system in patients with type 1 diabetes. *JAMA* 2016; **316**(13): 1407-8.
13. Ruan Y, Thabit H, Leelarathna L, et al. Variability of insulin requirements over 12 weeks of closed-loop insulin delivery in adults with type 1 diabetes. *Diabetes Care* 2016; **39**(5): 830-2.
14. Kovatchev B, Cobelli C, Renard E, et al. Multinational study of subcutaneous model-predictive closed-loop control in type 1 diabetes mellitus: summary of the results. *J Diabetes Sci Technol* 2010; **4**(6): 1374-81.
15. Hovorka R, Kumareswaran K, Harris J, et al. Overnight closed loop insulin delivery (artificial pancreas) in adults with type 1 diabetes: crossover randomised controlled studies. *BMJ* 2011; **342**: d1855.
16. Del Favero S, Bruttomesso D, Di Palma F, et al. First use of model predictive control in outpatient wearable artificial pancreas. *Diabetes Care* 2014; **37**(5): 1212-5.
17. Phillip M, Battelino T, Atlas E, et al. Nocturnal glucose control with an artificial pancreas at a diabetes camp. *N Engl J Med* 2013; **368**(9): 824-33.
18. FDA News Release. FDA approves first automated insulin delivery device for type 1 diabetes.http://www.fda.gov/NewsEvents/Newsroom/PressAnnouncements/ucm522974.htm (last accessed 24 October 2016).
19. Seaquist ER, Anderson J, Childs B, et al. Hypoglycemia and diabetes: a report of a workgroup of the American Diabetes Association and the Endocrine Society. *Diabetes Care* 2013; **36**(5): 1384-95.
20. Hovorka R, Shojaee-Moradie F, Carroll PV, et al. Partitioning glucose distribution/transport, disposal, and endogenous production during IVGTT. *Am J Physiol Endocrinol Metab* 2002; **282**(5): E992-1007.
21. Wilinska ME, Chassin LJ, Acerini CL, Allen JM, Dunger DB, Hovorka R. Simulation environment to evaluate closed-loop insulin delivery systems in type 1 diabetes. *J Diabetes Sci Technol* 2010; **4**(1): 132-44.
22. Strauss A, Corbin J. B*asics of qualitative research: grounded theory procedures and techniques. London Sage* 1990.

For peer review only

23. Leelarathna L, Dellweg S, Mader JK, et al. Day and night home closed-loop insulin delivery in adults with type 1 diabetes: three-center randomized crossover study. *Diabetes Care* 2014; **37**(7): 1931-7.
24. Tauschmann M, Allen JM, Wilinska ME, et al. Day-and-night hybrid closed-loop insulin delivery in adolescents with type 1 diabetes: A free-living, randomized clinical trial. *Diabetes Care* 2016; **39**(7): 1168-74.
25. Tauschmann M, Allen JM, Wilinska ME, et al. Home use of day-and-night hybrid closed-loop insulin delivery in suboptimally controlled adolescents with type 1 diabetes: a 3-week, free-living, randomized crossover trial. *Diabetes Care* 2016; **39**(11): 2019-25.
26. Bally L, Thabit H, Kojzar H, et al. Closed-loop insulin delivery in well-controlled type 1 diabetes adults: a free-living day-and-night randomised crossover study. *Lancet Diabetes Endocrinol* 2017. http://dx.doi.org/10.1016/S2213-8587(17)30001-3.

For peer review only

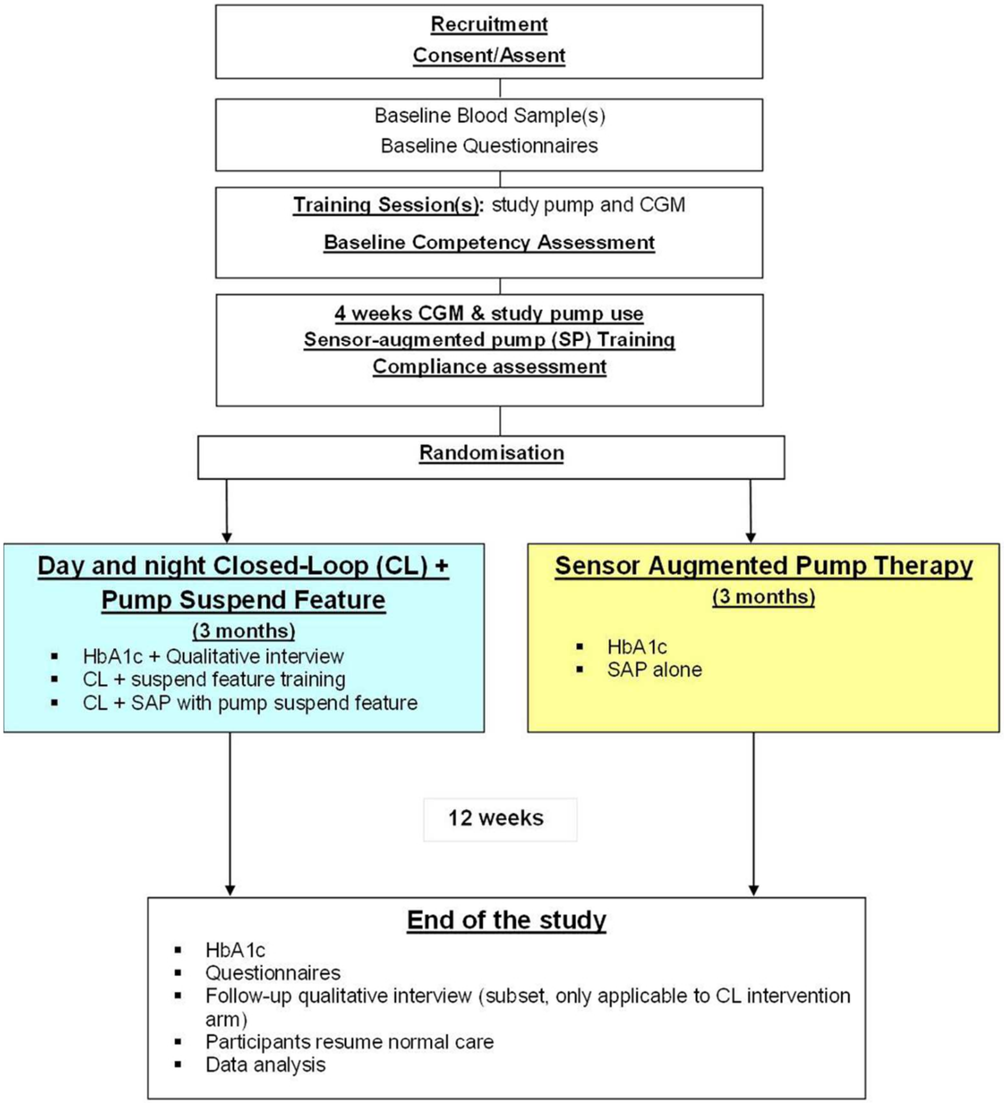

86x103mm (300 x 300 DPI)

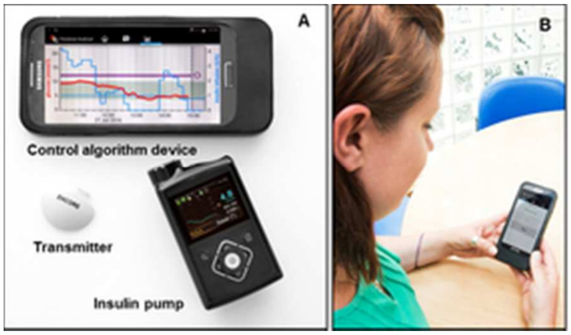

24x13mm (300 x 300 DPI)

peer review only

APPENDIX 1

Data Safety Monitoring Board

Dr Irl Hirsch (Chair), University of Washington School of Medicine, Seattle, WA, USA

Dr Michael Tansey (Member), University of Iowa Health Care, IA, USA

Dr Carol Levy (Member), Mount Sinai Hospital, New York, NY, USA

Study Management Team

Chief Investigator

Dr Roman Hovorka, University of Cambridge, Cambridge, UK

Principal Clinical Investigators (Youth)

Dr David Dunger, University of Cambridge, Cambridge, UK

Dr Daniela Elleri, Royal Hospital for Sick Children, Edinburgh, UK

Dr Fiona Campbell, St James's University Hospital, Leeds, UK

Dr Amy Criego, International Diabetes Center, Minneapolis, MN, USA

Principal Clinical Investigators (Adult)

Dr Mark Evans, University of Cambridge, Cambridge, UK

Dr Lalantha Leelarathna, Central Manchester University Hospitals NHS Foundation Trust, Manchester, UK

Dr Richard Bergenstal, International Diabetes Center, Minneapolis, MN, USA

Dr Viral Shah, University of Colorado Denver, Aurora, CO, USA

Clinical Investigators

Dr Carlo L Acerini, University of Cambridge, Cambridge, UK

Dr Hood Thabit, University of Cambridge, Cambridge, UK

Dr Martin Tauschmann, University of Cambridge, Cambridge, UK

Dr Lia Bally, University of Cambridge, Cambridge, UK

Study Co-ordinator (UK)

Mrs Josephine Hayes, University of Cambridge, Cambridge, UK

Study Co-ordinator (USA)

Dr Roy Beck, Jaeb Center for Health Research, Tampa, FL, USA

Medical Sociologist

Dr Julia Lawton, University of Edinburgh, Edinburgh, UK

For peer review only

Study Statistician

Dr Craig Kollman, Jaeb Center for Health Research, Tampa, FL, USA

Study Data Manager

Carlos Murphy, Jaeb Center for Health Research, Tampa, FL, USA

Study Sponsor (UK)

Cambridge University Hospitals NHS Foundation Trust, jointly with University of Cambridge, UK

Study Sponsor (USA)

Jaeb Center for Health Research, Tampa, FL, USA

Funder

JDRF

For peer review only

UNIVERSITY OF CAMBRIDGE

Cambridge University Hospitals NHS
NHS Foundation Trust

IMS Institute of Metabolic Science

Patient ID Label:

Centre NRES number: ....

Patient identification number for this trial: …………………….

For peer review only

CONSENT FORM

(Adults and Youth aged 16 and over)

Title of Project: Home testing of day and night closed loop with pump suspend feature

Name of Researcher: ....

Please initial box

1. I confirm that I have read and understood the information sheet version 2.1 dated 17/11/2015 for the above study and have had the opportunity to consider the information and I am satisfied with the answers I have been given.

2. I understand that my participation is voluntary and that I am free to withdraw at any time, without giving any reason, without my medical care or legal rights being affected in any way.

3. I give consent for the taking of blood and urine samples.

4. If I want to change my mind in the future and withdraw my consent for this study, then I understand that, if I request it, the samples will be destroyed.

5. I give consent to my GP being informed of my participation in this study.

6. I give consent to the sharing of anonymised research data arising from this study with other study partners in Europe and USA.

7. I give consent to anonymised blood samples being sent to USA for analysis.

8. If I choose to communicate with the research team via email, I acknowledge the associated risks as outlined in the information sheet.

9. I understand that relevant sections of my medical notes and data collected during the study may be looked at by individuals from the NHS Trust and regulatory bodies for audit or monitoring purposes.

10. If randomised to the closed loop study arm:

    a. I agree to participate in two qualitative interviews and for my contact details to be given to the study psychologist.

    b. I agree to have my family member/partner contacted by the study psychologist and to have their contact details forwarded.

    c. I agree for my interviews to be digitally recorded.

    d. I understand that anonymised quotes may be used in reports and publications.

11. I agree to return the study devices at the end of the study or earlier if consent is withdrawn.

12. I understand that in the unlikely event that study devices are not returned, legal measures may be undertaken, as a last resort after all other options have been exhausted, to recover the study devices.

13. I agree to take part in the study, and to follow the study team's instructions for the purposes of the study.

For peer review only

14. I give consent for my personal data to be kept by the clinical research team for up to 5 years after termination of the study so that they may contact me in the future with results of this and other similar studies. ☐

Name of participant	Date	Signature
Name of Person taking consent (if different from researcher)	Date	Signature
Researcher	Date	Signature

Copies: *1 for volunteer; 1 for researcher; 1 to be kept with hospital notes*

For peer review only

SPIRIT

STANDARD PROTOCOL ITEMS: RECOMMENDATIONS FOR INTERVENTIONAL TRIALS

SPIRIT 2013 Checklist: Recommended items to address in a clinical trial protocol and related documents*

Section/item	ItemNo	Description	Page number
**Administrative information**			
Title	1	Descriptive title identifying the study design, population, interventions, and, if applicable, trial acronym	1
Trial registration	2a	Trial identifier and registry name. If not yet registered, name of intended registry	2
	2b	All items from the World Health Organization Trial Registration Data Set	
Protocol version	3	Date and version identifier	5
Funding	4	Sources and types of financial, material, and other support	16
Roles and responsibilities	5a	Names, affiliations, and roles of protocol contributors	1, 16
	5b	Name and contact information for the trial sponsor	13
	5c	Role of study sponsor and funders, if any, in study design; collection, management, analysis, and interpretation of data; writing of the report; and the decision to submit the report for publication, including whether they will have ultimate authority over any of these activities	16
	5d	Composition, roles, and responsibilities of the coordinating centre, steering committee, endpoint adjudication committee, data management team, and other individuals or groups overseeing the trial, if applicable (see Item 21a for data monitoring committee)	14
**Introduction**			4

For peer review only

Background and rationale	6a	Description of research question and justification for undertaking the trial, including summary of relevant studies (published and unpublished) examining benefits and harms for each intervention	3
	6b	Explanation for choice of comparators	3
Objectives	7	Specific objectives or hypotheses	3
Trial design	8	Description of trial design including type of trial (eg, parallel group, crossover, factorial, single group), allocation ratio, and framework (eg, superiority, equivalence, noninferiority, exploratory)	5
**Methods: Participants, interventions, and outcomes**			
Study setting	9	Description of study settings (eg, community clinic, academic hospital) and list of countries where data will be collected. Reference to where list of study sites can be obtained	5, 7, 8
Eligibility criteria	10	Inclusion and exclusion criteria for participants. If applicable, eligibility criteria for study centres and individuals who will perform the interventions (eg, surgeons, psychotherapists)	5, 6
Interventions	11a	Interventions for each group with sufficient detail to allow replication, including how and when they will be administered	8, 9
	11b	Criteria for discontinuing or modifying allocated interventions for a given trial participant (eg, drug dose change in response to harms, participant request, or improving/worsening disease)	9
	11c	Strategies to improve adherence to intervention protocols, and any procedures for monitoring adherence (eg, drug tablet return, laboratory tests)	7, 8
	11d	Relevant concomitant care and interventions that are permitted or prohibited during the trial	8

Outcomes	12	Primary, secondary, and other outcomes, including the specific measurement variable (eg, systolic blood pressure), analysis metric (eg, change from baseline, final value, time to event), method of aggregation (eg, median, proportion), and time point for each outcome. Explanation of the clinical relevance of chosen efficacy and harm outcomes is strongly recommended	11, 12
Participant timeline	13	Time schedule of enrolment, interventions (including any run-ins and washouts), assessments, and visits for participants. A schematic diagram is highly recommended (see Figure)	7, 8, 18, 19, 20
Sample size	14	Estimated number of participants needed to achieve study objectives and how it was determined, including clinical and statistical assumptions supporting any sample size calculations	13
Recruitment	15	Strategies for achieving adequate participant enrolment to reach target sample size	5, 13
**Methods: Assignment of interventions (for controlled trials)**			
Allocation:			
Sequence generation	16a	Method of generating the allocation sequence (eg, computer-generated random numbers), and list of any factors for stratification. To reduce predictability of a random sequence, details of any planned restriction (eg, blocking) should be provided in a separate document that is unavailable to those who enrol participants or assign interventions	7
Allocation concealment mechanism	16b	Mechanism of implementing the allocation sequence (eg, central telephone; sequentially numbered, opaque, sealed envelopes), describing any steps to conceal the sequence until interventions are assigned	7
Implementation	16c	Who will generate the allocation sequence, who will enrol participants, and who will assign participants to interventions	5, 7

Blinding (masking)	17a	Who will be blinded after assignment to interventions (eg, trial participants, care providers, outcome assessors, data analysts), and how	N/A
	17b	If blinded, circumstances under which unblinding is permissible, and procedure for revealing a participant's allocated intervention during the trial	N/A
**Methods: Data collection, management, and analysis**			
Data collection methods	18a	Plans for assessment and collection of outcome, baseline, and other trial data, including any related processes to promote data quality (eg, duplicate measurements, training of assessors) and a description of study instruments (eg, questionnaires, laboratory tests) along with their reliability and validity, if known. Reference to where data collection forms can be found, if not in the protocol	8, 10, 12
	18b	Plans to promote participant retention and complete follow-up, including list of any outcome data to be collected for participants who discontinue or deviate from intervention protocols	9, 12
Data management	19	Plans for data entry, coding, security, and storage, including any related processes to promote data quality (eg, double data entry; range checks for data values). Reference to where details of data management procedures can be found, if not in the protocol	14
Statistical methods	20a	Statistical methods for analysing primary and secondary outcomes. Reference to where other details of the statistical analysis plan can be found, if not in the protocol	10, 11, 12, 13
	20b	Methods for any additional analyses (eg, subgroup and adjusted analyses)	12, 13
	20c	Definition of analysis population relating to protocol non-adherence (eg, as randomised analysis), and any statistical methods to handle missing data (eg, multiple imputation)	12, 13
**Methods: Monitoring**			

Data monitoring	21a	Composition of data monitoring committee (DMC); summary of its role and reporting structure; statement of whether it is independent from the sponsor and competing interests; and reference to where further details about its charter can be found, if not in the protocol. Alternatively, an explanation of why a DMC is not needed	13
	21b	Description of any interim analyses and stopping guidelines, including who will have access to these interim results and make the final decision to terminate the trial	13
Harms	22	Plans for collecting, assessing, reporting, and managing solicited and spontaneously reported adverse events and other unintended effects of trial interventions or trial conduct	12
Auditing	23	Frequency and procedures for auditing trial conduct, if any, and whether the process will be independent from investigators and the sponsor	14
**Ethics and dissemination**			
Research ethics approval	24	Plans for seeking research ethics committee/institutional review board (REC/IRB) approval	14, 15
Protocol amendments	25	Plans for communicating important protocol modifications (eg, changes to eligibility criteria, outcomes, analyses) to relevant parties (eg, investigators, REC/IRBs, trial participants, trial registries, journals, regulators)	15
Consent or assent	26a	Who will obtain informed consent or assent from potential trial participants or authorised surrogates, and how (see Item 32)	5, 7
	26b	Additional consent provisions for collection and use of participant data and biological specimens in ancillary studies, if applicable	N/A
Confidentiality	27	How personal information about potential and enrolled participants will be collected, shared, and maintained in order to protect confidentiality before, during, and after the trial	14

For peer review only - http://bmjopen.bmj.com/site/about/guidelines.xhtml

Declaration of interests	28	Financial and other competing interests for principal investigators for the overall trial and each study site	16
Access to data	29	Statement of who will have access to the final trial dataset, and disclosure of contractual agreements that limit such access for investigators	14
Ancillary and post-trial care	30	Provisions, if any, for ancillary and post-trial care, and for compensation to those who suffer harm from trial participation	N/A
Dissemination policy	31a	Plans for investigators and sponsor to communicate trial results to participants, healthcare professionals, the public, and other relevant groups (eg, via publication, reporting in results databases, or other data sharing arrangements), including any publication restrictions	15
	31b	Authorship eligibility guidelines and any intended use of professional writers	N/A
	31c	Plans, if any, for granting public access to the full protocol, participant-level dataset, and statistical code	N/A
**Appendices**			
Informed consent materials	32	Model consent form and other related documentation given to participants and authorised surrogates	3, 4, 5
Biological specimens	33	Plans for collection, laboratory evaluation, and storage of biological specimens for genetic or molecular analysis in the current trial and for future use in ancillary studies, if applicable	N/A

*It is strongly recommended that this checklist be read in conjunction with the SPIRIT 2013 Explanation & Elaboration for important clarification on the items. Amendments to the protocol should be tracked and dated. The SPIRIT checklist is copyrighted by the SPIRIT Group under the Creative Commons "Attribution-NonCommercial-NoDerivs 3.0 Unported" license.

For peer review only
